# *Gymnadenia conopsea* orchid: a systematic review

**DOI:** 10.3389/fphar.2025.1595714

**Published:** 2025-08-06

**Authors:** Jing-Yi Wu, Ruo-Nan Tang, Jia-Wen Wang, Wan-Ying Chen, Xing Liu, Ji-Wen Wang, Mei-Ya Li, Fu-Sheng Jiang

**Affiliations:** ^1^College of Life Sciences, Zhejiang Chinese Medical University, Hangzhou, Zhejiang, China; ^2^ Zhejiang-Hong Kong Joint Laboratory of Liver and Spleen Simultaneous Treatment in Traditional Chinese Medicine, College of Life Sciences, Zhejiang Chinese Medical University, Hangzhou, Zhejiang, China; ^3^ Shanghai Hope-tec Biotechnology Inc., Shanghai, China; ^4^Academy of Chinese Medical Sciences, Zhejiang Chinese Medical University, Hangzhou, Zhejiang, China

**Keywords:** *Gymnadenia conopsea*, phytochemistry, pharmacological activity, symbiotic germination, sustainable conservation

## Abstract

**Background:**

*Gymnadenia conopsea* (L.) R. Br., a medicinally significant orchid used for millennia in China, is systematically reviewed regarding its botany, resources, ethnomedicinal applications, phytochemistry, pharmacology, and propagation strategies to advance therapeutic utilization and conservation.

**Methods:**

Using keywords such as “*G. conopsea*,” “phytochemistry,” “propagation and breeding,” “bioactive compounds,” “immunomodulatory effects,” and “neuroprotective potential,” we systematically searched literature related to *G. conopsea* plants from databases including Web of Science, SciFinder, PubMed, ACS Publications, CNKI, Wanfang Data, Google Scholar, and Baidu Scholar.

**Results:**

A total of 1,074 papers were retrieved and 133 full-text articles were ultimately selected and comprehensively reviewed. Up to now, over 203 metabolites have been identified in the tubers of *G. conopsea*, including benzyl ester glucosides, stilbenoids, phenanthrenes, phenolic derivatives, alkaloids and polysaccharides. Pharmacological studies validate its multi-target therapeutic potential across tonification, anti-fatigue interventions, oxidative stress mitigation, antiviral defense, and management of gastric ulcers and silicosis. Despite extensive research on the pharmacological properties of crude extracts, the relationship between specific bioactive compounds and their corresponding pharmacological activities, particularly *in vivo*, remains poorly understood. Critically, overexploitation and habitat degradation have led to its classification as an endangered species. Current propagation efforts face significant challenges, including low natural germination rates, and dependence on specific habitats and obligate mycorrhizal fungi, precluding the development of efficient large-scale cultivation and seedling production systems.

**Conclusion:**

Marked progress has been made in characterizing small-molecule metabolites of *G. conopsea*, yet comprehensive structural elucidation of polysaccharides remains incomplete. Additionally, research must be intensified on synergistic interactions of bioactive constituents, molecular targets, mechanisms of action, and *in vivo* metabolic pathways to facilitate development of a quality standard system. For propagation, wild-simulated cultivation should be adopted for resource conservation, while optimizing symbiotic germination techniques is critical to overcome propagation bottlenecks, ultimately enabling sustainable utilization.

## Highlights

• This review contributes to updating the botany, traditional uses, resource status, phytochemistry, and pharmacology of *Gymnadenia conopsea.*
• The article further elaborates on the methodologies and challenges associated with the propagation and breeding of *G. conopsea.*

## 1 Introduction

*Gymnadenia conopsea* (L.) R. Br. commonly known as the palmate orchid, Tibetan notoginseng, Wangla, or Buddha’s hand orchid, is a species of the genus *Gymnadenia* R. Br. within the Orchidaceae family, characterized as a perennial botanical drug. Among the 27 species within the genus (Bateman et al., 2021a), five are endemic to China: *G. conopsea* (L.) R.Br., *G.orchidis* Lindl., *G.crassinervis* Finet, *G. bicornis* Tang and K. Y. Lang and *G.emeiensis* K.Y. Lang, predominantly found in the southwestern region of China (Xue, 2023). The morphology of *G. conopsea* is distinctive, with a plant height that can reach up to 60 cm, predominantly featuring pink flowers, although some individuals may exhibit pinkish-white blossoms. *G. orchidis* is relatively shorter, reaching a maximum height of approximately 35 cm, with flowers that are primarily purplish or pink, and occasionally white. *Gymnadenia crassinervis* can grow to a height of 55 cm, with flowers mainly pink and some slightly tinged with white. *G. bicornis* has a height range of 50–70 cm and presents flowers of a pale yellowish-green color, which are smaller in comparison to other species. Lastly, *G. emeiensis* has a height range of 30–50 cm and is notable for its white flowers.

The genus inhabits montane grasslands and semi-open woodlands (Brandrud et al., 2019), spanning temperate Eurasia to central India, including China, Japan, and the Himalayas (Bateman, 2021b; Anghelescu et al., 2023). In China, *G. conopsea* primarily occurs in Tibet, Qinghai, and Sichuan (Cha et al., 2024). Furthermore, *G. orchidis* is distributed in Qinghai, Shaanxi, Hubei, Gansu, etc.; *G. bicornis*, indigenous to Tibet, constitutes a distinctive species within the region; *G*. *crassinervis*, which is endemic to China, is located in Tibet, Yadong, Sichuan, and Yunnan. *G*. *emeiensis* primarily harvested in Mount Emei, Sichuan Province, originates in shrubbery-grassland habitats and boasts medicinal properties akin to that of *G. conopsea* (Xue, 2023). With the exception of *G. emeiensis*, the tubers of the other three species are frequently employed as substitutes for *G. conopsea* in Tibetan medicine. In this traditional medical system, *G. conopsea* is prescribed for conditions such as renal insufficiency, impotence, chronic pain, and urinary disorders due to its reputed yang-tonifying and essence-replenishing properties (Peng K. Z. et al., 2021). Modern studies validate its neuroprotective (anti-Alzheimer’s) (Luo, 2021), anti-oxidant properties (Yu L. et al., 2024), anti-fatigue (Liu et al., 2022), immunomodulatory (Yu, 2024b), and nootropic (Guo et al., 2022) activities. Over 200 metabolites, including glucosides, phenanthrenes, aromatic compounds, alkaloids, polysaccharides, lignans, flavones, triterpenoids, and steroids, have been isolated and characterized from the tubers of *G. conopsea*. Despite its therapeutic potential, the artificial cultivation on a large scale has not yet been achieved for this typical orchidaceae plant due to underdeveloped seeds, the challenges of natural reproduction, and its stringent habitat requirements. In recent years, the burgeoning market demand has spurred a proliferation of disorganized harvesting, leading to a drastic decline in *G. conopsea* resources, and the species is now teetering on the brink of extinction. Currently, recognized as a valuable medicinal substance, *G. conopsea* has been classified as an endangered species in both the “China Red List of Species” and the “IUCN Red List of Endangered Species”. Additionally, it has been designated as a second-class rare and endangered medicinal plant under the Convention on International Trade in Endangered Species of Wild Fauna and Flora (CITES) (Cheng et al., 2024a; Yu Y. P. et al., 2024).

This treatise will delineate the advancements in the study of *G. conopsea*, encompassing its botanical characteristics, traditional applications, phytochemical composition, pharmacological properties, and breeding. It aims to furnish a comprehensive reference for the rational and sustainable management, utilization, and conservation of *G. conopsea* resources in future endeavors.

## 2 Materials and methods

A comprehensive literature search was conducted across major scientific databases, including Web of Science, SciFinder, PubMed, ACS Publications, CNKI, Wanfang Data, Google Scholar, and Baidu Scholar, to identify studies pertaining to the phytochemical constituents, biological activities, ethnomedicinal applications, propagation, and breeding of *G. conopsea* (L.) R. Br. The search employed keywords such as “*G. conopsea*,” “phytochemistry,” “propagation and breeding,” “traditional uses,” “bioactive compounds,” “antioxidant activity,” “immunomodulatory effects,” and “neuroprotective potential,” utilizing Boolean operators (AND, OR) to optimize the search strategy. The scope was limited to literature published up to the end of 2024. Inclusion criteria encompassed peer-reviewed English articles specifically investigating *G. conopsea*’s phytochemistry, pharmacological properties, propagation, and breeding; relevant Chinese dissertations were also included to ensure broad representativeness. Non-peer-reviewed sources and studies unrelated to its medicinal or biological significance were excluded.

Systematic data extraction was performed on the selected studies, focusing on identified phytochemical constituents—including benzyl glucoside esters, stilbenoids, phenanthrenes, phenolic derivatives, alkaloids, polysaccharides, lignans, flavonoids, triterpenoids, and steroids—and their associated bioactivities, such as tonic effects, anti-fatigue interventions, mitigation of oxidative stress, antiviral defense, and therapeutic applications for gastric ulcers and silicosis. Notably, the isolation and characterization of dozens of novel compounds significantly expanded the known phytochemical profile of this species beyond previous literature (Shang et al., 2017; Meng et al., 2023). All compound structures presented in this review were meticulously drawn using ChemDraw Ultra 8.0 (PerkinElmer Inc., Waltham, MA, United States), facilitating the understanding of pharmacological activities and structure-activity relationships and promoting the future establishment of standardized quality control for *G. conopsea*.

Significant progress has been achieved in the isolation and characterization of *G. conopsea* metabolites, particularly small-molecule compounds. While further isolation and identification of trace novel structures contribute to enriching natural product libraries, they offer minimal insight into elucidating the traditional pharmacological significance of this medicinal orchid. Consequently, diverging from existing reviews (Shang et al., 2017; Meng et al., 2023), this study critically addresses research limitations and emphasizes the following imperatives: Standardized characterization of bioactive constituents with established traditional pharmacological relevance; Investigation of synergistic interactions among high-abundance efficacy components with confirmed bioactivity; Application of advanced methodologies to elucidate primary molecular targets and mechanisms of action for recognized active metabolites; collectively enabling comprehensive development of a quality standard system for *G. conopsea*. Critically, severe propagation constraints necessitate systematic research on wild resource domestication and artificial cultivation. This review examines key challenges and proposes: (1) Control of nematode infestations; (2) Implementation of semi-wild cultivation; (3) Supplementation with beneficial mycorrhizal fungi and recreation of conducive soil microenvironments to conserve germplasm resources; (4) Optimization of symbiotic germination technology to resolve seedling production bottlenecks. Therefore, this study synthesizes current knowledge on *G. conopsea*’s chemical composition, pharmacological activities, and resource status, identifies persistent challenges, and proposes strategic research directions to advance therapeutic applications and species conservation.

## 3 Study selection

**FIGURE 1:**
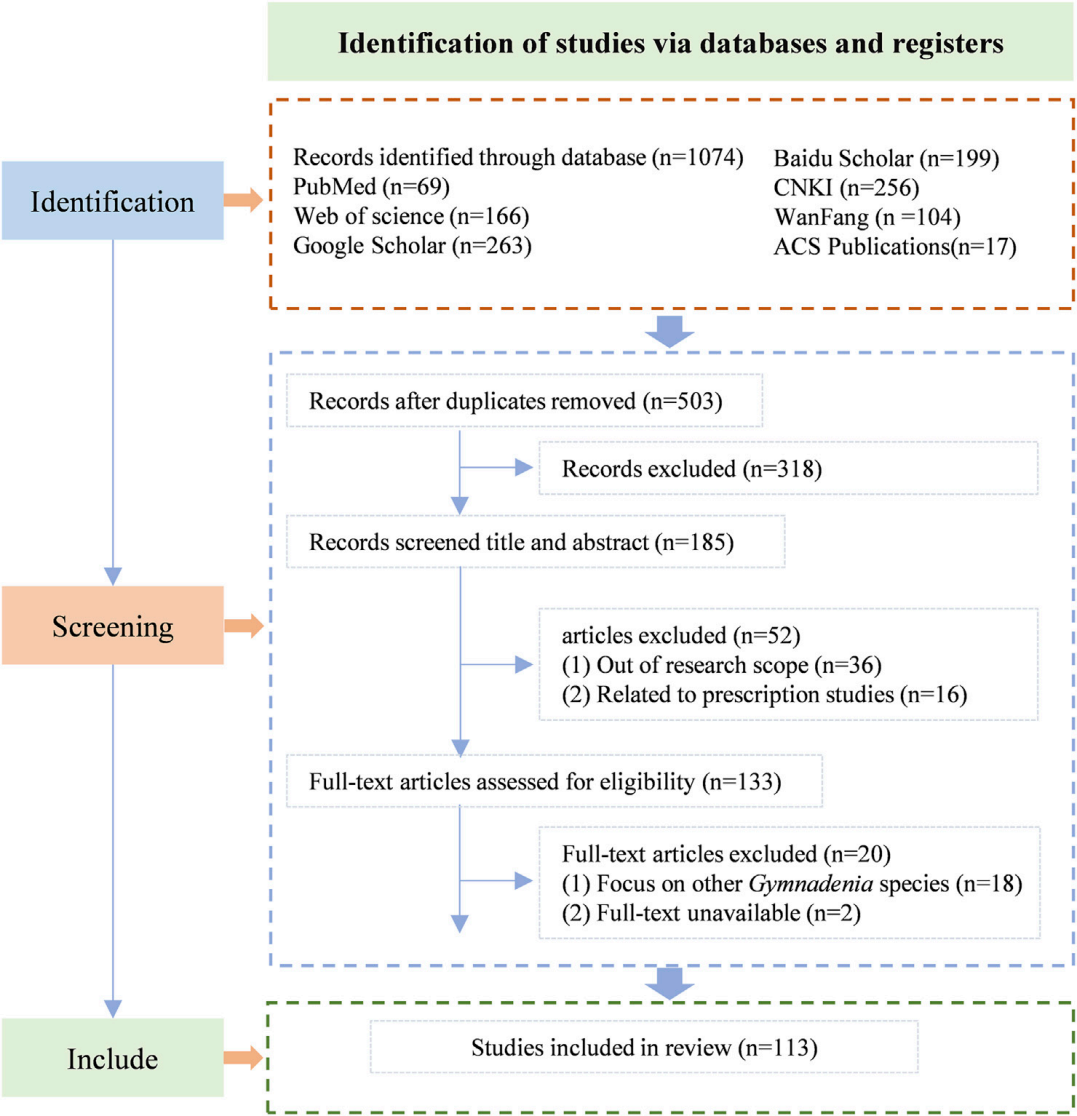
Flow diagram for the process of included studies identification (Page et al., 2021).

A systematic search was conducted across six major scientific databases, yielding a total of 1,074 records. The screening process followed the PRISMA flowchart framework adapted from Page et al. (Page et al., 2021) with modifications (Figure 1). The databases included Web of Science (n = 166), PubMed (n = 69), Google Scholar (n = 263), ACS Publications (n = 17), CNKI (n = 256), Wanfang Data (n = 104), and Baidu Scholar (n = 199). After removing duplicates using NoteExpress and conducting manual screening, 503 articles were retained for preliminary evaluation. Though title and abstract screening 318 records were excluded. The remaining 185 articles underwent full-text assessment. Among these, 36 articles were excluded due to being outside the research scope, 16 were related to prescription studies, 18 focused on other *Gymnadenia* species, and 2 had unavailable full-text versions. Following this rigorous selection process, 113 studies met all predefined inclusion criteria and were included in the qualitative synthesis.

## 4 Botanical characteristics and conservation status

*G. conopsea*, a perennial terrestrial and aromatic orchidaceous botanical drug, is commonly encountered within high-altitude forests, grasslands, shrublands, and inundated meadow regions at altitudes ranging from 0 to 4,700 m (Gao et al., 2020). It displays a marked preference for habitats with ample sunlight and flourishes predominantly on calcareous or alkaline substrates that are nutrient-poor (spanning from oligotrophic to mesotrophic conditions) (Meekers et al., 2012; Anghelescu et al., 2024). The natural population size of *G. conopsea* is small. It is mostly distributed sporadically or in sporadic patches in local areas of some counties in the Qinghai-Tibet Plateau (Yang et al., 2018a; Chen et al., 2023). Its abundance is inextricably linked to altitude, manifesting the unique characteristics of the plateau monsoon climate (Chen J. Y. et al., 2022; Yu Y. P. et al., 2024). Despite the existence of approximately 69 synonyms for this taxon, only *G. conopsea* (L.) R. Br. is widely recognized and accepted. This species typically attains a height of 20–60 cm and possesses slightly flattened roots. The lower section of the plant is usually divided into 3 to 5 digitate lobes, resembling a palm, and measures approximately 5 cm in length and 4 cm in diameter (Long et al., 2019). The stem is erect, cylindrical, and slender, adorned with 4 longitudinal leaves or oblique, and is rooted at the base with 2-3 brown, cylindrical sheaths, while the upper part bears 4-5 leaves and terminates in 1 to several bracteolate leaflets. The bracts are green, frequently tinged with purple at the margins, and are lanceolate with acuminate apices. The leaf blades are green, linear-lanceolate, narrowly oblong, or ribbon-like in morphology, with the lower leaves being erect to slightly spreading, measuring 5.5–15 cm in length and 1-2 (−2.5) cm in width, characterized by an acuminate or slightly obtuse apex, entire margins, a keel-like midrib, and a base that narrows into a clasping sheath. The racemes are densely flowered, cylindrical, and measure 5.5–15 cm in length, exhibiting a coloration that progresses from pale pink to lavender (rarely white or bright magenta). They reach full bloom in July-August, emitting an intense fragrance. The bracts are lanceolate, erect, and the apex is prolonged into a caudate shape, usually exceeding the length of the flower and ovary. The flowers are fragrant, predominantly pink, though occasionally pale pink to whitish-pink. The ovary is fusiform, with a slightly recurved apex, measuring approximately 8 mm in length including the peduncle. The pollen mass is ovoid in shape, characterized by a delicate pedicel and a mucilaginous disc. The mucilaginous disc assumes a linear-lanceolate morphology. The median sepal is broadly elliptical, measuring 3.5–5 mm in length and 3–4 mm in breadth. The apex is faintly saccate and marked by the presence of three veins. The lateral sepals are obliquely ovate, reflexed, and slightly exceed or are almost equivalent in length to the median sepal. The margins are incurved, and the apex terminates in an acute point, also traversed by three veins. The petals are vertically oriented, obliquely ovate-triangular in shape, corresponding in length to the median sepal and nearly matching the breadth of the lateral sepals. The margins are finely dentate, culminating in an acute apex. It is broadly cuneate-obovate and has three veins. The labellum projects anteriorly and exhibits a broadly obovate shape, with a length of 4–5 mm (Shang et al., 2017). Capsule, trigonal long cylindrical, sessile, ranging from 0.6 to 1.4 cm in length. The seeds are light brown, very numerous and small (Yang, 2018b). Each capsule contains approximately 8,000 to 10,000 seeds. Individual seeds measure approximately 450 μm in length. Scanning electron microscopy reveals that mature *G. conopsea* seeds exhibit a fusiform shape with surface ornamentation. The central portion of the seed houses the embryo, which is approximately 200 μm wide. Additionally, one end of the seed features an aperture measuring about 80 μm in width (Gao et al., 2019).

Under natural conditions, the asexual reproduction coefficient of *Gymnadenia* species is notably low. The growth and development of the hand-shaped tubers in the present year are contingent upon the nutritional transfer from the tubers of the preceding year; that is, the growth of a new tuber is maintained by consuming the old ones. This is obviously insufficient to meet the demands of large-scale cultivation. Furthermore, as an orchidaceous plant dependent upon a specific habitat, the germination of its seeds is contingent upon particular mycorrhizal fungi, thereby rendering the process arduous and resulting in an exceedingly low rate of natural germination (Shi, 2023a). Moreover, in the contemporary era, robust market demand, excessive exploitation, and the degradation of indigenous habitats have precipitated a drastic decline in the wild resources of *G. conopsea*, prompting its classification as an endangered species. Consequently, there is an imperative need to conduct comprehensive research on *G. conopsea* and to harness its resources efficiently.

## 5 Traditional uses

*G. conopsea* (commonly known as hand orchid), a traditional ethnomedicine with a millennium-long history of application, is characterized by a sweet-bitter taste, neutral nature, and heavy, greasy, soft, dilute, and pure properties. It primarily targets the lung, spleen, and stomach meridians, exhibiting therapeutic effects such as invigorating yang, consolidating essence, and nourishing vitality (Shi Y. et al., 2022). For decades, *G. conopsea* has been documented in the Pharmacopoeia of the People’s Republic of China as a key ingredient in multiple formulations, extensively utilized in Tibetan and Mongolian medical systems for kidney tonification and pulmonary disease management. Additionally, its significance as an aromatic orchid species has attracted research attention in European countries (Lin et al., 2020; Meng et al., 2023). The tuber of *G. conopsea* is predominantly employed in traditional botanical drug practices across Asia, including China, Nepal, and Japan (Lin et al., 2020; Meng et al., 2023). In China and Russia, its preparations—tinctures and Galenical formulations—are clinically prescribed for treating impotence and alleviating bronchial asthma symptoms, respectively (Nazim et al., 2001; Devkota et al., 2022).

In China, *G. conopsea* is categorized as a tonic botanical drug, with its medicinal use first recorded in *Tibetan Pharmacopoeia Somaratsa* (Moon King’s Medicinal Diagnoses), an ancient Tibetan medical text (Pe-Ru Ta-Na, 1985). *Yutuobencao* further elaborates its efficacy in dispelling cold, treating rheumatic disorders (Tibetan: Huangshui disease), delaying senescence, and promoting semen production (Gyu-Thogrnying-Mayon-Tanmgon-Po, 2006). *Dumubencao* describes its sweet and greasy taste, highlighting its role as a longevity-enhancing elixir (Santaraksita, 2006). *Blue Beryl (Vaidurya Sngon Po)*, a classical Tibetan medical treatise, emphasizes its capacity to strengthen the body, enhance male fertility, detoxify, and address spleen-related disorders when combined with brown sugar (Mao et al., 2012). The Tibetan Medicinal Materials Standard of Qinghai Province (Vol. 1) identifies *G. orchidis* (Tibetan: Wangla) as a common Tibetan botanical drug with applications in chronic debility, prolonged hemorrhage, chronic diarrhea, pulmonary deficiency-induced cough, and impotence (Administration, 2019).

According to the Tibetan Formula Database, *G. conopsea* tubers are included in 104 out of 4,500 traditional Tibetan prescriptions (2.3%), with 33 formulations targeting physical strengthening and anti-aging, 26 for kidney diseases, 12 for gout and musculoskeletal pain, 11 for pulmonary conditions, 7 for ophthalmic disorders, and the remainder for parasitic infections and miscellaneous ailments (Shang et al., 2017).

## 6 Phytochemistry

The research on the chemical constituents of *G. conopsea* primarily focuses on its tuberous part. To date, a total of 203 metabolites have been isolated and identified (Tables 1, 2). Among these, glycosides represent the most abundant class of chemical constituents. Additionally, stilbene derivatives, phenanthrenes, aromatic compounds, alkaloids, polysaccharides, lignans, flavones, triterpenoids, steroids, and other compounds have also been isolated and documented (Zi et al., 2010). The diverse array of chemical metabolites found in *G. conopsea* provides a substantial material basis for its various pharmacological activities. A comprehensive review of these metabolites is instrumental in deepening our understanding of the pharmacological mechanisms underlying *G. conopsea* and serves as an important reference for investigating the active metabolites present in other species within this genus.

**TABLE 1:** Glucosides isolated and identified from *G. conopsea*.

No.	Chemical name	Nucleus	R	R_1_	R_2_	R_3_	R_4_	Part of plant	Identification methods	Reference
1	loroglossin	A	OH	*β*-D-glc	H	*β*-D-glc		tubers	HPLC; FAB-MS; CC; NMR; FTIR; MPLC; TLC; linear gradient counter-current chromatography combined with elution-extrusion mode	Morikawa et al. (2006), Li et al. (2007a), Zi et al. (2008a), Yue et al. (2010), Feng et al. (2024)
2	militarine	A	H	*β*-D-glc	H	*β*-D-glc		tubers	NMR; MS; FTIR; CC; MPLC; HPLC; TLC; linear gradient counter-current chromatography combined with elution-extrusion mode	Li et al. (2007a), Zi et al. (2008a), Yue et al. (2010), Feng et al. (2024)
3	dactylorhin B	A	OH	*β*-D-glc	*β*-D-glc	*β*-D-glc		tubers	NMR; MS; FTIR; CC; MPLC; HPLC; TLC; linear gradient counter-current chromatography combined with elution-extrusion mode	Li et al. (2007a), Zi et al. (2008a), Yue et al. (2010), Feng et al. (2024)
4	dactylorhin A	A	H	*β*-D-glc	*β*-D-glc	*β*-D-glc		tubers	NMR; MS; FTIR; CC; MPLC; HPLC; TLC; linear gradient counter-current chromatography combined with elution-extrusion mode	Li et al. (2007a), Zi et al. (2008a), Yue et al. (2010), Feng et al. (2024)
5	coelovirins A	B	*β*-D-glc	OH	CH(CH_3_)_2_	H	OH	tubers	FTIR; CC; MPLC; HPLC; NMR; TLC	Zi et al. (2008a), Yue et al. (2010)
6	coelovirins B	B	*β*-D-glc	H	OH	OH	CH(CH_3_)_2_	tubers	NMR; MS; FTIR; CC; MPLC; HPLC; TLC	Li et al. (2008), Zi et al. (2008a), Yue et al. (2010)
7	coelovirins D	B	*β*-D-glc	H	OH	Oglc	CH(CH_3_)_2_	tubers	FTIR; CC; MPLC; HPLC; NMR; TLC	Zi et al. (2008a), Yue et al. (2010)
8	coelovirins E							tubers	FTIR; CC; MPLC; HPLC; NMR; TLC	Zi et al. (2008a), Yue et al. (2010)
9	dactylorhin E	C	H	H	*β*-D-glc	*β*-D-glc		tubers	NMR; MS; FTIR; CC; MPLC; HPLC; TLC	Li et al. (2008), Zi et al. (2008a), Yue et al. (2010)
10	coelovirins F	D	OH					tubers	NMR; MS; FTIR	Li et al. (2008)
11	coelovirins G	D	H					tubers	NMR; MS; FTIR	Li et al. (2008)
12	gymnoside I	C	H	H	H	*β*-D-glc		tubers	HPLC; NMR; FAB-MS; CC FTIR	Morikawa et al. (2006), Li et al. (2008)
13	gymnoside II	E	H	*β*-D-glc	H			tubers	HPLC; FAB-MS; NMR; CC	Morikawa et al. (2006)
14	gymnoside III	F	H	H	Ac			tubers	HPLC; FAB-MS; NMR; CC	Morikawa et al. (2006)
15	gymnoside IV	F	Cin	H	H			tubers	HPLC; FAB-MS; NMR; CC	Morikawa et al. (2006)
16	gymnoside V	F	H	Cin	H			tubers	HPLC; FAB-MS; NMR; CC	Morikawa et al. (2006)
17	gymnoside VI	F	H	H	Cin			tubers	HPLC; FAB-MS; NMR; CC	Morikawa et al. (2006)
18	gymnoside VII	F	Cin	H	Ac			tubers	HPLC; FAB-MS; NMR; CC	Morikawa et al. (2006)
19	gymnoside VIII	F	Ac	H	Ac			tubers	HPLC; FAB-MS; NMR; CC	Morikawa et al. (2006)
20	gymnoside IX	F	Ac	Cin	H			tubers	HPLC; FAB-MS; NMR; CC	Morikawa et al. (2006)
21	gymnoside X	F	Ac	*cis*-Cin	H			tubers	HPLC; FAB-MS; NMR; CC	Morikawa et al. (2006)
22	(−)-(2R,3S)-1-(4-β-D-glucopyranosyloxybenzyl)-2-O-β-D-glucopyranosyl-4-{4-[α-D-glucopyranosyl-(1 → 4)-β-D-glucopyranosyloxy]benzyl}-2-isobutyltartrate	A	OH	*β*-D-glc (4→1) α-D-glc	*β*-D-glc	*β*-D-glc		tubers	HPLC; MPLC; NMR; ESIMS; HRESIMS; TLC; CC	Zi et al. (2008a)
23	(−)-(2R,3S)-1-(4-β-D-glucopyranosyloxybenzyl)-2-O-β-D-glucopyranosyl-4-{4-[β-D-glucopyranosyl-(1 → 3)-β-D-glucopyranosyloxy]benzyl}-2-isobutyltartrate	A	OH	*β*-D-glc (3→1) α-D-glc	*β*-D-glc	*β*-D-glc		tubers	HPLC; MPLC; NMR; ESIMS; HRESIMS; TLC; CC	Zi et al. (2008a)
24	(−)-(2R,3S)-1-{4-[β-D-Glucopyranosyl-(1 → 3)-β-D-glucopyranosyloxy]benzyl}-2-O-β-D-glucopyranosyl-4-(4-β-D-glucopyranosyloxybenzyl)-2-isobutyltartrate	A	OH	*β*-D-glc	glc	*β*-D-glc (3→1) α-D-glc		tubers	HPLC; MPLC; NMR; ESIMS; HRESIMS; TLC; CC	Zi et al. (2008a)
25	(−)-(2R,3S)-1-(4-β-D-glucopyranosyloxybenzyl)-4-{4-[β-D-glucopyranosyl-(1 → 6)-β-D-glucopyranosyloxy]benzyl}-2-isobutyltartrate	A	OH	*β*-D-glc (6→1) α-D-glc	H	*β*-D-glc		tubers	HPLC; MPLC; NMR; ESIMS; HRESIMS; TLC; CC	Zi et al. (2008a)
26	(−)-(2R,3S)-1-(4-β-D-glucopyranosyloxybenzyl)-4-methyl-2-isobutyltartrate	C	OH	CH_3_	H	*β*-D-glc		tubers	HPLC; MPLC; NMR; ESIMS; HRESIMS; TLC; CC	Zi et al. (2008a)
27	(−)-(2R)-2-O-β-D-glucopyranosyl-4-(4-β-D-glucopyranosyloxybenzyl)-2-isobutylmalate	E	H	*β*-D-glc	*β*-D-glc			tubers	HPLC; MPLC; NMR; ESIMS; HRESIMS; TLC; CC	Zi et al. (2008a)
28	(−)-4-[β-D-glucopyranosyl-(1 → 4)- β-D-glucopyranosyloxy]benzyl alcohol	G	*β*-D-glc (4→1) *β*-D-glc	H				tubers	FTIR; CC; MPLC; HPLC; NMR; TLC; ESIMS; HRESIMS	Zi et al. (2008a), Yue et al. (2010)
29	(+)-4-[α-D-glucopyranosyl-(1 → 4) -β-D-glucopyranosyloxy]benzyl alcohol	G	*β*-D-glc (4→1) α-D-glc	H				tubers	FTIR; CC; MPLC; HPLC; NMR; TLC; ESIMS; HRESIMS	Zi et al. (2008a), Yue et al. (2010)
30	(−)-4-[β-D-glucopyranosyl-(1 → 3)- β-D-glucopyranosyloxy]benzyl alcohol	G	*β*-D-glc (3→1) *β*-D-glc	H				tubers	FTIR; CC; MPLC; HPLC; NMR; TLC; ESIMS; HRESIMS	Zi et al. (2008a), Yue et al. (2010)
31	(−)-4-[β-D-glucopyranosyl-(1 → 3)- β-D-glucopyranosyloxy]benzyl ethyl ether	G	*β*-D-glc (4→1) *β*-D-glc	CH_2_CH_3_				tubers	FTIR; CC; MPLC; HPLC; NMR; TLC; ESIMS; HRESIMS	Zi et al. (2008a), Yue et al. (2010)
32	dactylorhin C							tubers	UPLC-Orbitrap-MS/MS	Wang et al. (2020)
33	grammatophylloside C							tubers	UPLC-Orbitrap-MS/MS	Wang et al. (2020)
34	(−)-(2R)-2-O-glucopyranosyl-(1 → 6)-glucopyranosyloxy-2-isobutylmalate	H	H	*β*-D-glc (4→1) *β*-D-glc				tubers	UPLC-HRMS/MS; EIC	Lin et al. (2021)
35	(−)-(2R,3S)-1-{[4-glucopyranosyl-(1 → 6)-glucopyranosyloxy]benzyl}-2-O-glucopyranosyl- 2-isobutyltartrate	E	OH	*β*-D-glc (6→1) *β*-D-glc	*β*-D-glc			tubers	UPLC-HRMS/MS; EIC	Lin et al. (2021)
36	(2R)-2-hydroxy-2-(2-methylpropyl) butanedioic acid	H	OH	H				tubers	UPLC-HRMS/MS; EIC	Lin et al. (2021)
37	marylaurencinoside E							tubers	UPLC-HRMS/MS; EIC	Lin et al. (2021)
38	(−)-(2R)-1-(4-glucopyranosyloxybenzyl)-2-O-glucopyranosyl-4-{[4-glucopyranosyl-(1 → 6)-glucopyranosyloxy]benzyl}-2-isobutylmalate	A	H	*β*-D-glc	*β*-D-glc	*β*-D-glc (3→1) *β*-D-glc		tubers	UPLC-HRMS/MS; EIC	Lin et al. (2021)
39	(−)-(2R,3S)-1-benzyloxyl-2-O-glucopyranosyloxyl-2-isobutyltartra	C	OH	H	*β*-D-glc	H		tubers	UPLC-HRMS/MS; EIC	Lin et al. (2021)
40	(−)-(2R,3S)-1-(4-(6-hydroxymethyl)-glucopyranosyloxybenzyl)-4-methyl-2-isobutyltartrate							tubers	UPLC-HRMS/MS; EIC	Lin et al. (2021)
41	(−)-(2R,3S)-1-{[4-Glucopyranosyl-6-benzyl]benzyl}-2-O-glucopyranosyl-4-(4-glucopyranosyloxybenzyl)-2-isobutyltartrate	I	OH	*β*-D-glc				tubers	UPLC-HRMS/MS; EIC	Lin et al. (2021)
42	(−)-(2R)-1-(4-glucopyranosyloxybenzyl) 4-(p-hydroxy) benzyl-2-isobutyltartrate	A	OH	*β*-D-glc	H	H		tubers	UPLC-HRMS/MS; EIC	Lin et al. (2021)
43	(−)-(2R,3S)-1-(4-glucopyranosyloxybenzyl)-2-O-glucopyranosyl-4-benzyl-2-isobutyltartrate	A	OH	*β*-D-glc	*β*-D-glc	H		tubers	UPLC-HRMS/MS; EIC	Lin et al. (2021)
44	(−)-(2R,3S)-1-(4-glucopyranosyloxybenzyl)-2-O-glucopyranosyl-4-{[4-glucopyranosyl-6-benzyl]benzyl}-2-isobutyltartrate	J	OH	*β*-D-glc				tubers	UPLC-HRMS/MS; EIC	Lin et al. (2021)
45	(−)-(2R)-1-benzyl-2-O-glucopyranosyl-2-isobutylmalate	E	H	H	*β*-D-glc			tubers	UPLC-HRMS/MS; EIC	Lin et al. (2021)
46	(−)-(2R)-1-(4-hydroxy)benzyl-4-(4-glucopyranosyloxybenzyl)-2-isobutyltartrate	A	OH	H	H	*β*-D-glc		tubers	UPLC-HRMS/MS; EIC	Lin et al. (2021)
47	(−)-(2R)-1-{[4-glucopyranosyl-6-benzyl]benzyl}-2-O-glucopyranosyl-4-(4-glucopyranosyloxybenzyl)-2-isobutylmalate	I	H	*β*-D-glc				tubers	UPLC-HRMS/MS; EIC	Lin et al. (2021)
48	(−)-(2R)-1-(4-glucopyranosyl-6-benzyl)-2-O-glucopyranosyl-4-benzyl-2-isobutylmalate	A	OH	*β*-D-glc	*β*-D-glc	H		tubers	UPLC-HRMS/MS; EIC	Lin et al. (2021)
49	(−)-(2R)-1-(4-glucopyranosyloxybenzyl)-2-O-glucopyranosyl-4-{[4-glucopyranosyl-6-benzyl]benzyl}-2-isobutylmalate	J	H	*β*-D-glc				tubers	UPLC-HRMS/MS; EIC	Lin et al. (2021)
50	(−)-(2R)-1-(4-hydroxy)benzyl-4-(4-glucopyranosyloxybenzyl)-2-isobutylmalate	A	H	*β*-D-glc	H	H		tubers	UPLC-HRMS/MS; EIC	Lin et al. (2021)
51	(−)-(2R)-1-(4-glucopyranosyloxybenzyl)-4-(4-hydroxy)benzyl-2-isobutylmalate	A	H	H	H	*β*-D-glc		tubers	UPLC-HRMS/MS; EIC	Lin et al. (2021)
52	(−)-(2R,3S)-1-(4-glucopyranosyloxybenzyl)-4-{[4-glucopyranosyl-6-benzyl]benzyl}-2-isobutyltartrate	I	OH	H				tubers	UPLC-HRMS/MS; EIC	Lin et al. (2021)
53	(−)-(2R,3S)-1-{[4-glucopyranosyl-6-benzyl]benzyl}-4-(4-glucopyranosyloxy-benzyl)-2-isobutyltartrate	J	OH	H				tubers	UPLC-HRMS/MS; EIC	Lin et al. (2021)
54	4-methoxymethylbenzyl-β-D-glucoside							tubers	HPLC; FAB-MS; NMR; CC	Morikawa et al. (2006)
55	bis (4-hydroxybenzyl)-ethermono-β-D-glucopyranoside							tubers	HPLC; FAB-MS; NMR; CC	Morikawa et al. (2006)
56	4-(β-D-glucopyranosyloxy) benzoic aldehyde							tubers	HPLC; MPLC; NMR; ESIMS; HRESIMS; TLC; CC	Zi et al. (2008a)
57	4-(β-D-glucopyranosyloxy)benzyl ethyl ether							tubers	HPLC; MPLC; NMR; ESIMS; HRESIMS; TLC; CC	Zi et al. (2008a)
58	phenyl-β-D-glucopyranoside							tubers	HPLC; FAB-MS; NMR; CC	Morikawa et al. (2006)
59	4-formylphenyl-β-D-glucopyranoside							tubers	HPLC; FAB-MS; NMR; CC	Morikawa et al. (2006)
60	benzyl-β-D-glucopyranoside							tubers	HPLC; FAB-MS; NMR; CC	Morikawa et al. (2006)
61	trans-ferulic acid-β-D-glucoside							tubers	HPLC; MPLC; NMR; ESIMS; HRESIMS; TLC; CC	Zi et al. (2008a)
62	cis-ferulic acid-β-D-glucoside							tubers	HPLC; MPLC; NMR; ESIMS; HRESIMS; TLC; CC	Zi et al. (2008a)
63	N6-(4-hydroxybenzyl)adenine riboside							tubers	HPLC; MPLC; NMR; ESIMS; HRESIMS; TLC; CC	Zi et al. (2008a)
64	daucosterol							tubers	NMR; MS; FTIR	Li et al. (2007a)
65	dioscin							tubers	CC; PTLC; EI-MS; FAB-MS; NMR; TLC	Li et al. (2001)
66	dactylose A							tubers	HPLC; FAB-MS; NMR; CC	Morikawa et al. (2006)
67	dactylose B							tubers	HPLC; FAB-MS; NMR; CC	Morikawa et al. (2006)
68	n-butyl-β-D-fructopyranose							tubers	CC; PTLC; EI-MS; FAB-MS; NMR; TLC	Li et al. (2001)
69	thymidine							tubers	HPLC; FAB-MS; NMR; CC	Morikawa et al. (2006)
70	4-hydroxybenzyl-β-D-glucopyranoside							tubers	HPLC; GC; FTIR	Yang (2010)
71	4-methylphenyl-β-D-glucopyranoside							tubers	HPLC; GC; FTIR	Yang (2010)
72	4-hydroxyphenyl-β-D-glucopyranoside							tubers	HPLC; GC; FTIR	Yang (2010)
73	2-hydroxy-1-[(4-hydroxyphenyl) methyl]-4-methylphenyl-1-β-D-glucopyranoside							tubers	CC; ODS CC; Sephadex LH-20 CC; semi-prep HPLC; NMR; HR-ESI-MS; XRD; IR; UV-Vis	Qin et al. (2024a)
74	2-hydroxy-5-methylphenyl-1-(4-β-D-glucopyranosyloxybenzyl)							tubers	CC; ODS CC; Sephadex LH-20 CC; semi-prep HPLC; NMR; HR-ESI-MS; XRD; IR; UV-Vis	Qin et al. (2024a)
75	(2S)-2-(β-D-glucopyranosyloxy)-2-(2-methylpropyl) butanedioic acid 4-methyl ester							tubers	CC; ODS CC; Sephadex LH-20 CC; semi-prep HPLC; NMR; HR-ESI-MS; XRD; IR; UV-Vis	Qin et al. (2024a)

Note: UPLC, ultra-high performance liquid chromatography; HESI, heated electrospray ionization; Orbitrap-MS/MS, orbitrap tandem mass spectrometry; NCE, normalized collision energy; CC, column chromatography; PTLC, preparative thin-layer chromatography; EI-MS, electron ionization mass spectrometry; FAB-MS, fast atom bombardment mass spectrometry; NMR, nuclear magnetic resonance spectroscopy; TLC, thin-layer chromatography; EIC, extracted ion chromatogram; UV-Vis, ultraviolet-visible spectroscopy; IR, infrared spectroscopy; XRD, single crystal X-ray diffraction; HR-ESI-MS, high-resolution electrospray ionizationmass spectrometry; NMR, nuclear magnetic resonance; Semi-Prep HPLC, semi-preparative high-performance liquid chromatography; Sephadex LH-20 CC, sephadex LH-20, column chromatography; ODS CC, octadecylsilyl column chromatography; GC-MS, gas chromatography-mass spectrometry; MPLC, medium-pressure liquid chromatography; HPLC, high-performance liquid chromatography; ESI-MS, electrospray ionization mass spectrometry; HPTLC, high-performance thin-layer chromatography; FTIR, fourier transform infrared spectroscopy.

**TABLE 2:** Stilbenes, phenanthrenes, aromatics, alkaloids, lignans, flavones, terpenoids and other compounds isolated and identified from *G. conopsea*.

No.	Name	Nucleus	R	R_1_	R_2_	R_3_	R_4_	R_5_	R_6_	Part of plant	Identification methods	Reference
Stilbenes												
76	batatacin Ш	K	H	OH	H	OCH_3_	H	OH	H	tubers	CC; HPLC; NMR; EIMS: UV-Vis/IR; TLC/HPTLC	Matsuda et al. (2004)
77	3′-0-methylbatatacin Ш	K	H	OH	H	OCH_3_	H	OCH_3_	H	tubers	CC; HPLC; NMR; EIMS: UV-Vis/IR; TLC/HPTLC	Matsuda et al. (2004)
78	3′,5-dihydroxy-2-(4-hydroxybenzyl)-3-methoxybibenzyl	K	CH_2_-C_6_H_4_-OH	OCH_3_	H	OH	H	OH	H	tubers	CC; HPLC; NMR; EIMS: UV-Vis/IR; TLC/HPTLC	Matsuda et al. (2004)
79	3,3′-dihydroxy-2-(4-hydroxybenzyl)-5-methoxybibenzyl	K	CH_2_-C_6_H_4_-OH	OH	H	OCH_3_	H	OH	H	tubers	CC; HPLC; NMR; EIMS: UV-Vis/IR; TLC/HPTLC	Matsuda et al. (2004)
80	gymeonopin D	K	CH_2_-C_6_H_4_-OH	OH	H	OCH_3_	H	OCH_3_	H	tubers	CC; HPLC; NMR; EIMS: UV-Vis/IR; TLC/HPTLC	Matsuda et al. (2004)
81	3,3′-dihydroxy-2,6-bis(4-hydroxybenzyl)-5-methoxybibenzyl	K	CH_2_-C_6_H_4_-OH	OH	H	OCH_3_	CH_2_-C_6_H_4_-OH	OH	H	tubers	CC; HPLC; NMR; EIMS: UV-Vis/IR; TLC/HPTLC	Matsuda et al. (2004)
82	5-O-methylbatatacin Ⅲ	K	H	OCH_3_	H	OCH_3_	H	OH	H	tubers	HPLC; GC-MS; CC	Li et al. (2006)
83	2-(4-hydroxybenzyl)-3′-O-methylbatataein Ⅲ	K	CH_2_-C_6_H_4_-OH	OCH_3_	H	OH	H	OCH_3_	H	tubers	HPLC; GC-MS; CC	Li et al. (2006)
84	arundinin	K	H	OH	CH_2_-C_6_H_4_-OH	OCH_3_	H	OH	H	tubers	HPLC; GC-MS; CC	Li et al. (2006)
85	arundin	K	CH_2_-C_6_H_4_-OH	OH	H	OCH_3_	CH_2_-C_6_H_4_-OH	H	H	tubers	HPLC; GC-MS; CC	Li et al. (2006)
86	bulboeodin C	K	CH_2_-C_6_H_4_-OH	OCH_3_	CH_2_-C_6_H_4_-OH	OH	H	OH	H	tubers	HPLC; GC-MS; CC	Li et al. (2006)
87	bulboeodin D	K	CH_2_-C_6_H_4_-OH	OH	CH_2_-C_6_H_4_-OH	OCH_3_	H	OH	H	tubers	HPLC; GC-MS; CC	Li et al. (2006)
88	gymconopin D	K	CH_2_-C_6_H_4_-OH	OH	H	OCH_3_	H	OCH_3_	H	tubers	CC; HPLC; NMR; EIMS: UV-Vis/IR; TLC/HPTLC	Matsuda et al. (2004)
89	isorhapontigenin	L	OH	OCH_3_	OH	OH				tubers	UPLC-Orbitrap-MS/MS	Wang et al. (2020)
90	rhaponticin	L	OCH_3_	OH	OH	glc				tubers	UPLC-Orbitrap-MS/MS	Wang et al. (2020)
91	piceatannol	L	OH	OH	OH	OH				tubers	UPLC-Orbitrap-MS/MS	Wang et al. (2020)
92	dihydroresveratrol	K	H	OH	H	OH	H	H	OH	tubers	UPLC-Orbitrap-MS/MS	Wang et al. (2020)
Phenanthrenes												
93	Gymconopins A	M	CH_2_-C_6_H_4_-OH	OCH_3_	H	OH	OH	H		tubers	CC; HPLC; NMR; EIMS: UV-Vis/IR; TLC/HPTLC	Matsuda et al. (2004)
94	Gymconopins B	M	H	OCH_3_	CH_2_-C_6_H_4_-OH	OH	OH	H		tubers	CC; HPLC; NMR; EIMS: UV-Vis/IR; TLC/HPTLC	Matsuda et al. (2004)
95	Gymconopins C									tubers	CC; HPLC; NMR; EIMS: UV-Vis/IR; TLC/HPTLC	Matsuda et al. (2004)
96	1-(4-hydrobenzyl)-4-methoxy-9,10-dihydrophenanthrene-2,7-diol	M	CH_2_-C_6_H_4_-OH	OH	H	OCH_3_	H	OH		tubers	CC; HPLC; NMR; EIMS: UV-Vis/IR; TLC/HPTLC	Matsuda et al. (2004)
97	l-(4-hydroxybenzyl)-4-methoxyphenanthrene-2,7-diol									tubers	CC; HPLC; NMR; EIMS: UV-Vis/IR; TLC/HPTLC	Matsuda et al. (2004)
98	2-methoxy-9,10-dihydrophenanthrene-4,5-diol	M	H	OCH_3_	H	OH	OH	H		tubers	CC; HPLC; NMR; EIMS: UV-Vis/IR; TLC/HPTLC	Matsuda et al. (2004)
99	4-methoxy-9,10-dihydrophenanthrene-2,7-diol	M	H	OH	H	OCH_3_	H	OH		tubers	CC; HPLC; NMR; EIMS: UV-Vis/IR; TLC/HPTLC	Matsuda et al. (2004)
100	blestriarene A									tubers	CC; HPLC; NMR; EIMS: UV-Vis/IR; TLC/HPTLC	Matsuda et al. (2004)
101	blestriarene B									tubers	UPLC–Orbitrap–MS/MS	Wang et al. (2020)
Aromatics												
102	phenol	N	H	OH	H	H				tubers	CC; PTLC; EI-MS; FAB-MS; NMR; TLC	Li et al. (2001)
103	eugenol	N	OCH_3_	OH	H	CH_2_-CH = CH_2_				tubers	CC; PTLC; EI-MS; FAB-MS; NMR; TLC	Li et al. (2001)
104	p-hydroxybenzyl alcohol	N	H	OH	H	CH_2_OH				tubers	FTIR; CC; MPLC; HPLC; NMR; TLC; MS	Yang et al. (2009), Yue et al. (2010)
105	vanillic acid	N	OCH_3_	OH	H	COOH				tubers	FTIR; CC; MPLC; HPLC; NMR; TLC; MS	Yang et al. (2009), Yue et al. (2010)
106	4 -methoxyphenyl β -D -glucopyranoside									tubers	HPLC; CC; MS; NMR; TLC	Yang et al. (2009)
107	p -hydroxybenzaldehyde	N	H	OH	H	CHO				tubers	FTIR; CC; MPLC; HPLC; NMR; TLC; MS	Yang et al. (2009), Yue et al. (2010)
108	4-methylphenyl β-D-glucopyranoside	N	H	Oglc	H	CH_3_				tubers	HPLC; CC; MS; NMR; TLC	Yang et al. (2009)
109	4-hydroxybenzyl β-D-glucopyranoside	N	H	CH_2_ Oglc	H	OH				tubers	HPLC; CC; MS; NMR; TLC	Yang et al. (2009)
110	4-(β-D-glucopyranosyloxyl)benzoic aldehyde	N	H	Oglc	H	CHO				tubers	FTIR; CC; MPLC; HPLC; NMR; TLC	Yue et al. (2010)
111	4-methoxybenzyl β-D-glucoside	N	H	CH_2_ Oglc	H	OCH_3_				tubers	FTIR; CC; MPLC; HPLC; NMR; TLC	Yue et al. (2010)
112	4-(β-D-glucopyranosyloxyl)benzyl ethyl ether	N	H	Oglc	H	CH_2_OCH_2_CH_3_				tubers	FTIR; CC; MPLC; HPLC; NMR; TLC	Yue et al. (2010)
113	bis(4-hydroxybenayl)-ether mono-β-D-glucoside									tubers	FTIR; CC; MPLC; HPLC; NMR; TLC	Yue et al. (2010)
114	trans-ferulic acid β-D-glucoside									tubers	FTIR; CC; MPLC; HPLC; NMR; TLC	Yue et al. (2010)
115	cis-ferulic acid β-D-glucoside									tubers	FTIR; CC; MPLC; HPLC; NMR; TLC	Yue et al. (2010)
116	3-hydroxybenzoic acid	N	OH	H	H	COOH				tubers	FTIR; CC; MPLC; HPLC; NMR; TLC	Yue et al. (2010)
117	4-hydroxyisophthalic acid	N	COOH	OH	H	COOH				tubers	FTIR; CC; MPLC; HPLC; NMR; TLC	Yue et al. (2010)
118	4-hydroxybenzyl alcohol	N	H	OH	H	CH_2_OH				tubers	FTIR; CC; MPLC; HPLC; NMR; TLC	Yue et al. (2010)
119	4-hydroxybenzylmethyl ether	N	H	OH	H	CH_2_OCH_3_				tubers	FTIR; CC; MPLC; HPLC; NMR; TLC	Yue et al. (2010)
120	4-hydroxybenzyl aldehyde	N	H	OH	H	CHO				tubers	FTIR; CC; MPLC; HPLC; NMR; TLC	Yue et al. (2010)
121	4-hydroxybenzoic acid	N	H	OH	H	CH_2_OCH_3_				tubers	FTIR; CC; MPLC; HPLC; NMR; TLC	Yue et al. (2010)
122	trans-p-hydroxyphenylpropenoic acid	N	H	OH	H	CHO				tubers	FTIR; CC; MPLC; HPLC; NMR; TLC	Yue et al. (2010)
123	cis-p-hydroxyphenylpropenoic acid	N	H	OH	H	COOH				tubers	FTIR; CC; MPLC; HPLC; NMR; TLC	Yue et al. (2010)
124	4-(ethoxymethyl) phenol	N	H	OH	H	CH_2_OCH_2_CH_3_				tubers	linear gradient counter-current chromatography combined with elution-extrusion mode	Feng et al. (2024)
125	ferulic acid	N	OCH_3_	OH	H	COOH				tubers	UPLC-Orbitrap-MS/MS	Wang et al. (2020)
126	isoferulic acid	N	OH	OCH_3_	H	CH = CHCOOH				tubers	UPLC-Orbitrap-M UPLC-Orbitrap-MS/MS S/MS	Wang et al. (2020)
127	dactylose B									tubers	UPLC-Orbitrap-MS/MS	Wang et al. (2020)
128	4-methoxyphenyl β-D-glucopyranoside									tubers	UPLC-Orbitrap-MS/MS	Wang et al. (2020)
129	(E)-4-methoxycinnamic acid	N	H	OCH_3_	H	CH = CHCOOH				tubers	UPLC-Orbitrap-MS/MS	Wang et al. (2020)
130	tremuloidin									tubers	UPLC-Orbitrap-MS/MS	Wang et al. (2020)
131	phenyl-3-deoxyheopyranoside									tubers	UPLC-HRMS/MS; EIC	Lin et al. (2021)
132	neochlorogenic acid									tubers	UPLC-HRMS/MS; EIC	Lin et al. (2021)
133	phenyl-O-glucopyranoside									tubers	UPLC-HRMS/MS; EIC	Lin et al. (2021)
Alkaloids												
134	cyclo(L-Leu-L-Tyr)									tubers	FTIR; CC; MPLC; HPLC; NMR; TLC	Yue et al. (2010)
135	cyclo(L-Leu-L-Pro)									tubers	FTIR; CC; MPLC; HPLC; NMR; TLC	Yue et al. (2010)
136	cyclo(L-Val-L-Tyr)									tubers	FTIR; CC; MPLC; HPLC; NMR; TLC	Yue et al. (2010)
137	cyclo(L-Ala-D-Phe)									tubers	FTIR; CC; MPLC; HPLC; NMR; TLC	Yue et al. (2010)
138	N-trans-feruloyltyramine	O	OCH_3_							tubers	FTIR; CC; MPLC; HPLC; NMR; TLC	Yue et al. (2010)
139	Cyclo[glycine-L-S-(4″-hydroxybenzyl)cysteine]									tubers	CC; MPLC; HPLC; NMR; 2D-NMR; ESI-MS/HR-ESI-MS; TLC	Zi et al. (2010)
140	cyclo(L-Val-D-Tyr)									tubers	CC; MPLC; HPLC; NMR; 2D-NMR; ESI-MS/HR-ESI-MS; TLC	Zi et al. (2010)
141	conopsamide A									tubers	MPLC; HPLC; NMR; 2D-NMR; 1D-NMR; ESIMS/HR-ESIMS	Lin et al. (2017)
142	6-quinolinecarboxylic acid									tubers	UPLC–Orbitrap–MS/MS	Wang et al. (2020)
143	trans-indole-3-acrylic acid									tubers	UPLC–Orbitrap–MS/MS	Wang et al. (2020)
144	befunolol									tubers	UPLC–Orbitrap–MS/MS	Wang et al. (2020)
145	cyclo(tyrosy-tyrosyl)									tubers	UPLC–Orbitrap–MS/MS	Wang et al. (2020)
146	cyclo(leucylprolyl)									tubers	UPLC–Orbitrap–MS/MS	Wang et al. (2020)
147	N-(4-hydroxybenzy) adenine riboside									tubers	UPLC–Orbitrap–MS/MS	Wang et al. (2020)
148	dibenzylamine									tubers	UPLC–Orbitrap–MS/MS	Wang et al. (2020)
149	(+)-chelidonine									tubers	UPLC–Orbitrap–MS/MS	Wang et al. (2020)
150	(2E)-3-(4-hydroxy-phenyl)-N-[2-(4-hydroxy-phenyl)-ethyl]-acrylamide	O	H							tubers	UPLC–Orbitrap–MS/MS	Wang et al. (2020)
151	2,3,4,9-tetrahydro-1H-β-carboline-3-carboxylic acid									tubers	UPLC–Orbitrap–MS/MS	Wang et al. (2020)
152	N-phenyl-2-naphthylamine									tubers	UPLC–Orbitrap–MS/MS	Wang et al. (2020)
153	N-(4-methyoxyphenyl)-1H-pyrazolo									tubers	UPLC–Orbitrap–MS/MS	Wang et al. (2020)
Lignans												
154	arctigenin									tubers	FTIR; CC; MPLC; HPLC; NMR; TLC	Yue et al. (2010)
155	lappaol A									tubers	FTIR; CC; MPLC; HPLC; NMR; TLC	Yue et al. (2010)
156	lappaol F									tubers	FTIR; CC; MPLC; HPLC; NMR; TLC	Yue et al. (2010)
157	erythro-Buddlenol E									tubers	FTIR; CC; MPLC; HPLC; NMR; TLC	Yue et al. (2010)
158	pinoresinol									tubers	UPLC–Orbitrap–MS/MS	Wang et al. (2020)
Flavones												
159	quercitin-3,7-di-O-β-D-glueopyranoside	P	Oglc	H	Oglc	OH	OH			tubers	CC; PTLC; EI-MS; FAB-MS; NMR; TLC	Li et al. (2001)
160	quercetin-3′-β-O -glucoside	P	OH	H	OH	Oglc	OH			tubers	UPLC–Orbitrap–MS/MS	Wang et al. (2020)
161	cirsimarin	P	OCH_3_	OCH_3_	H	H	Oglc			tubers	UPLC–Orbitrap–MS/MS	Wang et al. (2020)
162	astragalin	P	OH	H	Oglc	H	OH			tubers	UPLC–Orbitrap–MS/MS	Wang et al. (2020)
163	kaempferol-7-O-glucoside	P	Oglc	H	OH	H	OH			tubers	UPLC–Orbitrap–MS/MS	Wang et al. (2020)
164	desmethylxanthohumol									tubers	UPLC–Orbitrap–MS/MS	Wang et al. (2020)
165	isorhamnetin	P	OH	H	OH	OCH_3_	OH			tubers	UPLC–Orbitrap–MS/MS	Wang et al. (2020)
166	naringenin chalcone									tubers	UPLC–Orbitrap–MS/MS	Wang et al. (2020)
167	equol									tubers	UPLC–Orbitrap–MS/MS	Wang et al. (2020)
168	galangin	P	OH	H	OH	H	H			tubers	UPLC–Orbitrap–MS/MS	Wang et al. (2020)
169	apigenin-7-O-glucoside	P	Oglc	H	H	H	OH			tubers	UPLC-HRMS/MS; EIC	Lin et al. (2021)
Terpenoids												
170	*β*-sitosterol	Q	OH							tubers	CC; PTLC; EI-MS; FAB-MS; NMR; TLC	Li et al. (2001)
171	*β*-daucosterin	Q	Oglc							tubers	NMR; MS; FTIR	Li et al. (2008)
172	mascaroside									tubers	UPLC–Orbitrap–MS/MS	Wang et al. (2020)
173	(±)-abscisic acid									tubers	UPLC–Orbitrap–MS/MS	Wang et al. (2020)
174	3β,6β,19α-trihydroxy-urs-12-en-28-oic acid									tubers	UPLC–Orbitrap–MS/MS	Wang et al. (2020)
175	5(10)-estrene-3β,17β-diol									tubers	UPLC–Orbitrap–MS/MS	Wang et al. (2020)
176	7α-methyl-5α-androstane-3β,11β,17β-triol									tubers	UPLC–Orbitrap–MS/MS	Wang et al. (2020)
177	lup-20(29)-en-28-al									tubers	UPLC–Orbitrap–MS/MS	Wang et al. (2020)
178	lupenone									tubers	UPLC–Orbitrap–MS/MS	Wang et al. (2020)
179	poriferasterol	R	OH							tubers	UPLC–Orbitrap–MS/MS	Wang et al. (2020)
180	4,4-dimethyl-5α-cholesta-8,14,24-trien-3β-ol									tubers	UPLC–Orbitrap–MS/MS	Wang et al. (2020)
181	lupeol									tubers	UPLC–Orbitrap–MS/MS	Wang et al. (2020)
182	(22E)-stigmasta-3,5,22-triene	R	H							tubers	UPLC–Orbitrap–MS/MS	Wang et al. (2020)
Other compounds												
183	tripalmitin									tubers	NMR; MS; FTIR	Li et al. (2008)
184	N-butyl-*β*-D-fructopyranoside									tubers	CC; PTLC; EI-MS; FAB-MS; NMR; TLC	Li et al. (2001)
185	4-hydroxyphenyl-4-O-glucopyranosyl-glucopyranoside									tubers	UPLC-HRMS/MS; EIC	Lin et al. (2021)
186	citric acid									tubers	UPLC–Orbitrap–MS/MS	Wang et al. (2020)
187	succinic acid									tubers	UPLC–Orbitrap–MS/MS	Wang et al. (2020)
188	benzyl-[(6-oxo-7,8,9,10-tetrahydro-6H-benzo[c]chromen-3yl)oxy]-acetate									tubers	UPLC–Orbitrap–MS/MS	Wang et al. (2020)
189	aloeresin A									tubers	UPLC–Orbitrap–MS/MS	Wang et al. (2020)
190	frangulin B									tubers	UPLC–Orbitrap–MS/MS	Wang et al. (2020)
191	cleomiscosin A									tubers	UPLC–Orbitrap–MS/MS	Wang et al. (2020)
192	bis-(methylbenzylidene)-sorbitol									tubers	UPLC–Orbitrap–MS/MS	Wang et al. (2020)
193	umbelliferone									tubers	UPLC–Orbitrap–MS/MS	Wang et al. (2020)
194	2-hydroxy-2-(4′-hydroxybenzyl)-4-methylcyclopent-4-ene-1,3-dione									tubers	CC; MPLC; HPLC; NMR; 2D-NMR; ESI-MS/HR-ESI-MS; TLC	Zi et al. (2010)
195	2-hydroxy-4-hydroxymethyl-3-(4′-hydroxyphenyl)cyclopent-2-enone									tubers	CC; MPLC; HPLC; NMR; 2D-NMR; ESI-MS/HR-ESI-MS; TLC	Zi et al. (2010)
196	(2R,3R,4S,5S,7S,8S,9S)-2,3,8,9-tetrahydroxy-7-methyl-pentaoxatetracyclo[6.6.2.0^4,5^.0^7,8^]hexadecane									tubers	CC; ODS CC; Sephadex LH-20 CC; semi-prep HPLC; NMR; HR-ESI-MS; XRD; IR; UV-Vis	Qin et al. (2024b)
197	(5S) -5- (hydroxymethyl) -4- [(E) - [5 '- (hydroxymethyl) furan-2' - yl] methylene] -2- [(Z) - (4 '' - hydroxyphenyl) methylene] tetrahydrofuran-3-one									tubers	CC; ODS CC; Sephadex LH-20 CC; semi-prep HPLC; NMR; HR-ESI-MS; XRD; IR; UV-Vis	Qin et al. (2024b)
198	5-hydroxymethyl-2-furaldehyde									tubers	CC; ODS CC; Sephadex LH-20 CC; semi-prep HPLC; NMR; HR-ESI-MS; XRD; IR; UV-Vis	Qin et al. (2024b)
199	bis-(5-formylfurfuryl) ether									tubers	CC; ODS CC; Sephadex LH-20 CC; semi-prep HPLC; NMR; HR-ESI-MS; XRD; IR; UV-Vis	Qin et al. (2024b)
200	pollenfuran A									tubers	CC; ODS CC; Sephadex LH-20 CC; semi-prep HPLC; NMR; HR-ESI-MS; XRD; IR; UV-Vis	Qin et al. (2024b)
201	pollenfuran B									tubers	CC; ODS CC; Sephadex LH-20 CC; semi-prep HPLC; NMR; HR-ESI-MS; XRD; IR; UV-Vis	Qin et al. (2024b)
202	5- ((4-O-β- D-glucopyranosylbenzyloxy) methyl) - furan-2-carboxaldehyde									tubers	CC; ODS CC; Sephadex LH-20 CC; semi-prep HPLC; NMR; HR-ESI-MS; XRD; IR; UV-Vis	Qin et al. (2024b)
203	9-p-hydroxybenzylhypoxanthine									tubers	CC; ODS CC; Sephadex LH-20 CC; semi-prep HPLC; NMR; HR-ESI-MS; XRD; IR; UV-Vis	Qin et al. (2024a)

Note: UPLC, Ultra-high performance liquid chromatography; HESI, heated electrospray ionization; Orbitrap-MS/MS: High-resolution tandem mass spectrometry; NCE, normalized collision energy; CC, column chromatography; PTLC, Preparative thin-layer chromatography; EI-MS, electron ionization mass spectrometry; FAB-MS, fast atom bombardment mass spectrometry; NMR, nuclear magnetic resonance spectroscopy; TLC, Thin-layer chromatography; EIC, extracted ion chromatogram; UV-Vis, Ultraviolet-Visible Spectroscopy; IR, infrared spectroscopy; XRD, Single Crystal X-ray Diffraction; HR-ESI-MS, High-Resolution Electrospray Ionization Mass Spectrometry; NMR, nuclear magnetic resonance; semi-prep HPLC, Semi-preparative high-performance liquid chromatography; Sephadex LH-20 CC, Sephadex LH-20, column chromatography; ODS CC, octadecylsilyl column chromatography; GC-MS, Gas chromatography-mass spectrometry; MPLC: Medium-pressure liquid chromatography; HPLC, High-performance liquid chromatography; NMR, nuclear magnetic resonance; ESI-MS, electrospray ionization mass spectrometry; HPTLC, High-performance thin-layer chromatography; FTIR, Fourier transform infrared spectroscopy.

### 6.1 Glucosides

**FIGURE 2:**
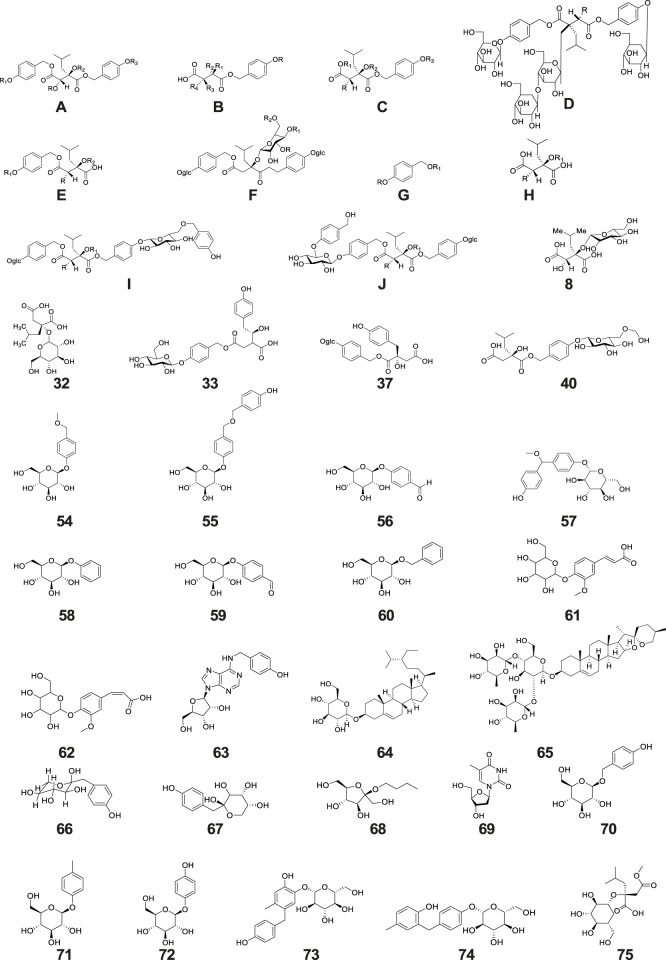
Structure of glucosides isolated and identified from *G. conopsea*.

Glucosides represent one of the paramount chemical constituents within the composition of *G. conopsea*, with a total of 73 distinct compounds having been rigorously investigated and successfully isolated (Table 1; Figure 2). Based on their structural configurations, these glucosides are categorizable into benzylester glucosides and additional varieties of glucosides.

#### 6.1.1 Benzylester glucosides

Fifty-four benzylester glucosides have been successfully isolated and identified from *G. conopsea*. The fundamental core structures are either 2-isobutyl tartaric acid or 2-isobutyl hydroxysuccinic acid. These acids integrate with one or more gluconyl benzyl alcohols to form a variety of complex compounds (Wang et al., 2020). According to the differences in their organic acids, they are classified as (2R, 3S)-2-isobutyl tartaric acid derivates and (2R)-2-isobutyl hydroxysuccinic acid derivatives. Li et al. (2007a) and Li et al. (2008) were the first to isolate nine benzylester glucosides (No. 1-4, 6, 9–12) from the ethanol extract of the tuber of *G. conopsea*. In 2006, Morikawa et al. (2006) isolated fourteen benzylester glucosides (No. 1-4, 12–21) from the methanol extraction of *G. conopsea* tuber. In addition, Zi et al. (2008a) also isolated eleven benzylester glucosides (No. 5-9, 22–27) from the ethanol extract of *G. conopsea* tuber. Furthermore, Yue et al. (2010) isolated and identified four new benzylester glucosides (No. 28–31) from the ethanolic extract of *G. conopsea* tuber. The research team headed by Wang et al. (2020) delineated two distinct compounds, dactylorhin C (No. 32) and grammatophylloside C (No. 33), from the 95% methanol extract of the *G. conopsea* tuber, utilizing UPLC-Orbitrap-MS/MS analytical techniques. Furthermore, an assortment of twenty benzylester glucosides (No. 34–53) was characterized from the ethanol extract of *G. conopsea* (Lin et al., 2021).

Benzylester glucosides represent a class of compounds found in the tubers of *Gymnadenia conopsea* (hand orchid) that are not only diverse in structural types but also often present in considerable concentrations. Li (2007b) quantified the content of five specific compounds—loroglossin (No. 1), militarine (No. 2), dactylorhin B (No. 3), dactylorhin A (No. 4), and dactylorhin E (No. 9)—in tubers collected from five distinct geographical regions: Lijiang (Yunnan), Tibet, Yuxian County (Hebei), Kangding (Sichuan), and Xining (Qinghai). The measured concentrations ranged from 0.25 to 3.06 mg/g, 0.10–0.56 mg/g, 0.67–4.073 mg/g, 0.51–2.33 mg/g, and 0.09–0.20 mg/g, respectively. Notably, samples from Xining, Qinghai exhibited the highest content for four of these compounds (No. 1, 2, 3, 4), with dactylorhin E (No. 9) ranking second highest. The total content of these five compounds reached 10.186 mg/g in the Xining samples, followed by the Tibetan samples at 5.458 mg/g. Similarly, He (2023a) employed HPLC to determine the content of three benzylester glucosides—loroglossin (No. 1), militarine (No. 2), and dactylorhin A (No. 4)—in ten batches of *G. conopsea* samples. The corresponding concentrations ranged from 0.30 to 12.7 mg/g, 0.09–1.76 mg/g, and 0.40–5.59 mg/g, respectively. These findings demonstrate significant variations in the content of these compounds among samples from different origins and batches. Consequently, there is an urgent need to implement standardized cultivation practices to mitigate quality variations arising from factors such as differences in regional soil conditions, climate, and the predominance of wild-sourced material (nearly all current samples).

#### 6.1.2 Other glucosides

A total of 22 other glucoside derivatives (No. 54–75) were successfully isolated and characterized from the tubers of *G. conopsea*, as demonstrated in Table 1 and Figure 2 (Li et al., 2001; Morikawa et al., 2006; Zi, 2008b; Yang, 2010; Qin et al., 2024a).

### 6.2 Stilbenes

**FIGURE 3:**
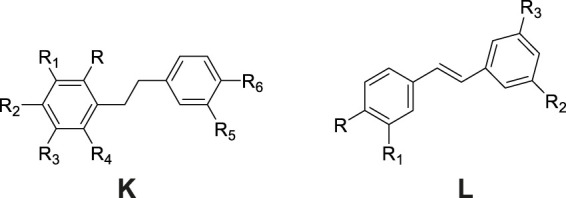
Structure of stilbene compounds isolated and identified from *G. conopsea*.

Stilbene derivatives constitute a specialized class of phytochemicals defined by a 1,2-diphenylethylene skeleton or its polymeric variants as structural backbones. The structural diversity arises from substitution patterns primarily occurring at positions 2, 3, 4, 5, 6, 3′, and 4′ (Figure 3), with hydroxyl (-OH), methoxy (-OCH_3_), glucosyloxy (-O-glc), and p-hydroxybenzyl groups being the predominant substituents (Table 2; Figure 3). Phytochemical investigations of *G. conopsea* have yielded significant findings: Li et al. (Li et al., 2006; Matsuda et al., 2004) systematically characterized thirteen stilbenoids (No. 76–88) from methanolic tuber extracts. Subsequent research by Wang et al. (Wang et al., 2020) expanded the chemical repertoire through the isolation of four novel derivatives from 95% methanol extracts, including isorhapontigenin (No. 89), rhaponticin (No. 90), piceatannol (No. 91), and dihydroresveratrol (No. 92).

### 6.3 Phenanthrenes

**FIGURE 4:**
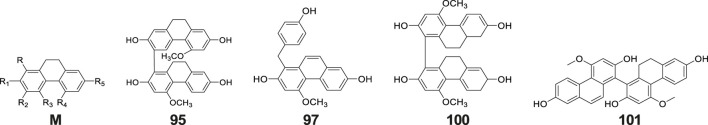
Structure of phenanthrenes compounds isolated and identified from *G. conopsea*.

Phenanthrenes represent a class of polycyclic aromatic hydrocarbons comprising three benzene rings, typically featuring dihydrophenanthrene as the fundamental structural core. The substituents are predominantly situated at the positions C-1, C-2, -3, -4, -5 and -7 (Figure 4). The prevalent substituents encompass OH, OCH_3_ or *p*-hydroxybenzyl. To date, nine phenanthrene derivatives have been isolated from *G. conopsea* and structurally characterized, including: gymconopins A-C (No. 93–95), 1-(4-hydrobenzyl)-4-methoxy-9,10-dihydrophenanthrene-2,7-diol (No. 96), l-(4-hydroxy benzyl)-4-methoxyphenanthrene-2,7-diol (No. 97), 2-methoxy-9,10- dihydrophenan threne-4,5-diol (No. 98), 4-methoxy-9,10-dihydrophenanthrene-2,7- diol (No. 99), blestriarene A (No. 100), and blestriarene B (No. 101) (Matsuda et al., 2004; Wang et al., 2020). These compounds exhibit variations in hydroxylation patterns and benzyl substitution, as detailed in Table 2 and Figure 4.

Bibenzyls and phenanthrenes represent characteristic constituents of Orchidaceae plants, extensively documented in *Dendrobium* species (Zhai et al., 2022), *Bletilla striata* (Jiang et al., 2021), and tubers of *G. conopsea* (Meng et al., 2023). Bibenzyl compounds undergo cytochrome P450-catalyzed oxidation to form dihydrophenanthrene intermediates, which subsequently undergo aromatization to yield phenanthrene scaffolds. These compounds, characterized by benzene rings bearing phenolic hydroxyl groups, are classified as plant polyphenols and demonstrate potent free radical-scavenging activities. They constitute key antioxidant metabolites in *G. conopsea* tubers (Table 4). In their investigation of radical-scavenging activities among phenanthrenes and bibenzyls isolated from *G. conopsea* tubers, Morikawa et al.(Morikawa et al., 2006) established critical structure-activity relationships: For phenanthrenes, dihydrogenation at positions 9 and 10 enhances bioactivity, while *p*-hydroxybenzyl substitution at C-1 or C-3 similarly potentiates activity; conversely, for bibenzyl derivatives, methylation at the 3′-position diminishes activity, whereas *p*-hydroxybenzyl substitution at C-2 and/or C-6 augments efficacy. These structural insights provide a valuable foundation for future applications in synthetic biology aimed at the targeted biosynthesis of high-activity compounds through rational modification of natural product scaffolds.

### 6.4 Aromatic compounds

**FIGURE 5:**
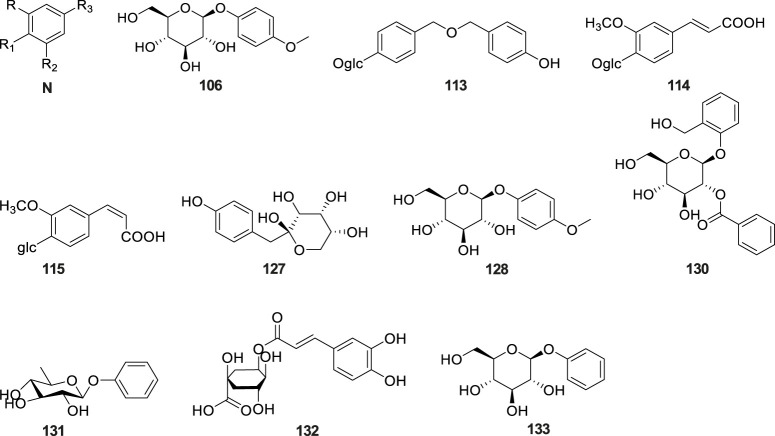
Structure of aromatic compounds isolated and identified from *G. conopsea*.

Phytochemical studies have revealed that phenolic compounds constitute the predominant class of aromatic constituents isolated from *G. conopsea* tubers. As ubiquitous secondary metabolites in the plant kingdom, these phenolic derivatives demonstrate significant biological relevance in both plant biochemistry and therapeutic applications. Systematic investigations have identified 32 structurally distinct phenolic derivatives from this orchid species (Table 2; Figure 5). Li et al. (2001) purified phenol (No. 102) and eugenol (No. 103) from an ethanol extract of *G. conopsea* tubers. Subsequently, Yang et al. (2009) utilized advanced techniques such as reverse-phase column chromatography to isolate and identify six additional compounds (No. 104–109) from the n-butanol extract of *G. conopsea* tubers. Furthermore, fourteen phenolic compounds (No. 110–123) were separated from the ethanol extract of *G. conopsea* tubers (Yue et al., 2010). Additionally, Feng et al. (2024) successfully isolated 4-(ethoxymethyl) phenol (No. 124) using linear gradient counter-current chromatography combined with elution-extrusion mode. Moreover, Wang et al. (2020) and Lin et al. (2021) expanded the phenolic profile through 95% methanol and ethanol extractions, yielding six (No. 125–130) and three (No. 131–133) additional compounds, respectively.

These phenolic metabolites are generally present at low levels in *G. conopsea* tubers, and virtually no reports exist on their *in vitro* or *in vivo* pharmacological activities. Future research should prioritize integrating the documented pharmacological effects of *G. conopsea* in traditional Chinese medicine with computational approaches—such as network pharmacology analysis and molecular docking techniques—to predict and validate the bioactivities of these compounds.

### 6.5 Alkaloids

**FIGURE 6:**
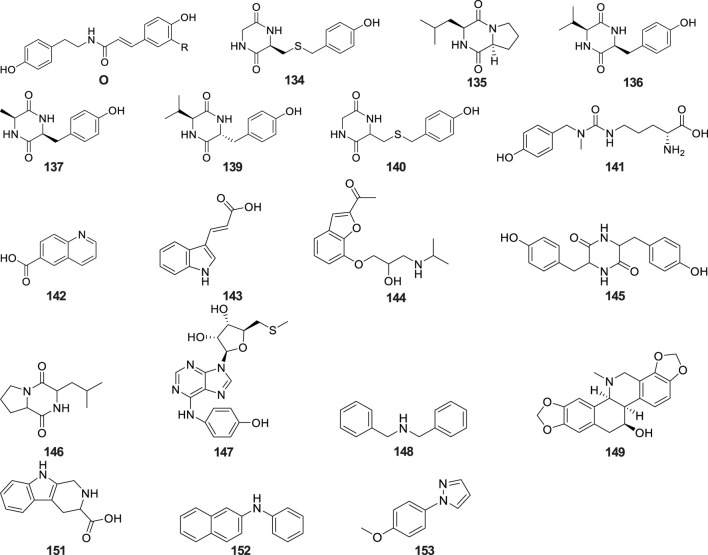
Structures of alkaloids isolated and identified from *G. conopsea*.

Alkaloids are a class of nitrogen-containing alkaline organic compounds with alkali-like properties, historically referred to as pseudoalkaloids due to their alkali-like characteristics. To date, 20 alkaloids have been isolated and identified from *G. conopsea* (Table 2; Figure 6). Yue et al. (2010) isolated and identified five alkaloids—cyclo(L-Leu-L-Tyr) (No. 134), cyclo(L-Leu-L-Pro) (No. 135), cyclo (L-Val-L-Tyr) (No. 136), cyclo(L-Ala-D-Phe) (No. 137) and N-*trans*-feruloyltyramine (No. 138) —from the ethanol extract of *G. conopsea* tubers. Three additional alkaloids, cyclo[glycine-L-S-(4′-hydroxybenzyl)cysteine] (No. 139), cyclo(L-Val- D-Tyr) (No. 140) (Zi et al., 2010) and conopsamide A (No. 141) were isolated from the tubers of *G. conopsea* (Lin et al., 2017). Furthermore, Wang et al. (2020) identified 12 more alkaloids (No. 142–153) from the 95% methanol aqueous solution of *G. conopsea* tubers.

### 6.6 Lignans

**FIGURE 7:**
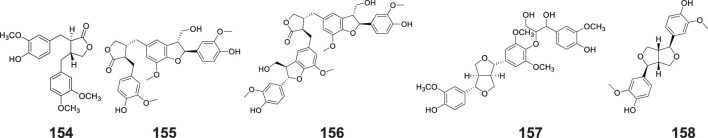
Structures of lignans isolated and identified from *G. conopsea*.

Lignans constitute a class of natural compounds that are synthesized through the dimerization of two phenylpropanoid (C_6_-C_3_) units. In their native state, these compounds predominantly occur as free aglycones rather than glycosidically bound forms. Within the phytochemical profile of *G. conopsea*, lignans exhibit limited distribution, with only five representatives currently documented (Table 2; Figure 7). Notably, phytochemical analysis of the tuber ethanol extract by Yue et al. (2010) identified four furanolignans: arctigenin (No. 154), lappaol A (No. 155), lappaol F (No. 156) and erythro-buddlenol E (No. 157), by Yue et al. (2010). Among these, the first three compounds demonstrated inhibitory effects on Fe^2+^-Cys-induced MDA formation in rat liver microsomes (Table 4). In a separate investigation, Wang et al. (2020) detected pinoresinol (No. 158) through targeted fractionation of tubers extracted with 95% methanol, marking the first identification of this tetrahydrofuran-type lignan in the genus. These lignans are characteristically abundant in *Arctium lappa* (burdock seeds) and exhibit diverse biological activities including antitumor, anti-inflammatory, and immunomodulatory effects (Yosri et al., 2023), suggesting their potential contribution to the pharmacological properties of *G. conopsea*.

### 6.7 Flavones

**FIGURE 8:**
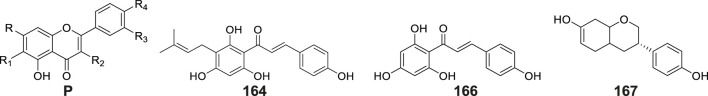
Structures of flavones isolated and identified from *G. conopsea*.

Flavonoids constitute a prominent class of phenylpropanoid derivatives featuring a characteristic C15 skeleton with two aromatic rings (A- and B-rings) interconnected by a heterocyclic pyran moiety (C-ring). These phytochemicals exhibit extensive structural plasticity through hydroxylation, glycosylation, and methylation modifications, contributing to their ecological roles and pharmacological potential. To date, a total of eleven flavonoids have been isolated and characterized from plants of the *Gymnadenia* species, six of which are glycoside compounds (Table 2; Figure 8). The isolation of quercetin-3,7-di-O-β-D-glucopyranoside (No. 159) was first reported by Li et al. (2001). Subsequent phytochemical profiling of *G. conopsea* tuber extracts by Wang et al. (2020) employing 95% aqueous methanol extraction led to the identification of nine derivatives: quercetin-3′-*β*-O-glucoside (No. 160), cirsimarin (No. 161), astragalin (No. 162), kaempferol-7-O-glucoside (No. 163), desmethylxanthohumol (No.164), isorhamnetin (No. 165), naringenin chalcone (No. 166), equol (No. 167) and galangin (No. 168). Furthermore, Lin et al. (2021) successfully elucidated apigenin-7-O-glucoside (No. 169) from the tubers of *G. conopsea*. While these flavonoids are established bioactive constituents in traditional Chinese medicines, their pharmacological contributions are concentration-dependent. Notably, quantitative data on their abundance in *G. conopsea* remain unreported in the literature.

### 6.8 Terpenoids and steroids

**FIGURE 9:**
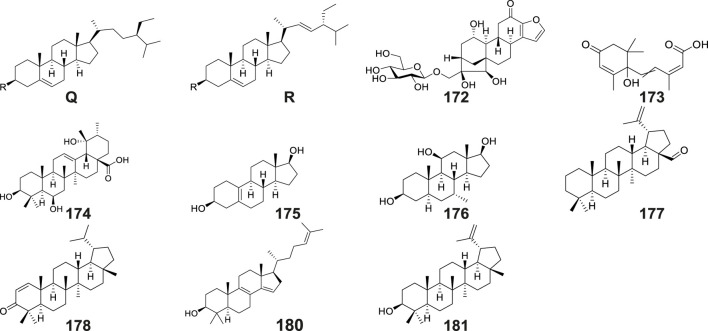
Structures of terpenoids and steroids isolated and identified from *G. conopsea*.

Terpenoids and their derivatives represent a class of secondary metabolites biosynthesized via the mevalonate pathway, featuring isoprene units (C5 units) as their structural backbones. Structurally distinct from terpenoids, steroids constitute a unique family of tetracyclic systems comprising three fused cyclohexane rings and one cyclopentane ring (cyclopentane-perhydrophenanthrene skeleton), which exhibit broad phylogenetic distribution across living organisms. Phytochemical investigations of *Gymnadenia* species have thus far elucidated 13 terpenoid and steroid derivatives (Table 2; Figure 9), including: *β*-sitosterol (No. 170) (Li et al., 2001), *β*-daucosterin (No. 171) (Li et al., 2008), mascaroside (No. 172), (±)-abscisic acid (No. 173), 3*β*,6*β*,19*α*-trihydroxy- urs-12-en- 28-oic acid (No. 174), 5(10)-estrene- 3*β*,17*β*-diol (No.175), 7*α*-methyl-5*α*- androstane- 3*β*,11*β*,17*β*-triol (176 No.), lup-20(29)-en-28-al (No.177), lupenone (No. 178), poriferasterol (No. 179), 4,4-dimethyl-5*α*-cholesta-8, 14,24-trien-3*β*-ol (No. 180), lupeol (No. 181), and (22E)-stigmasta-3,5,22-triene (No. 182) (Wang et al., 2020).

### 6.9 Other compounds

**FIGURE 10:**
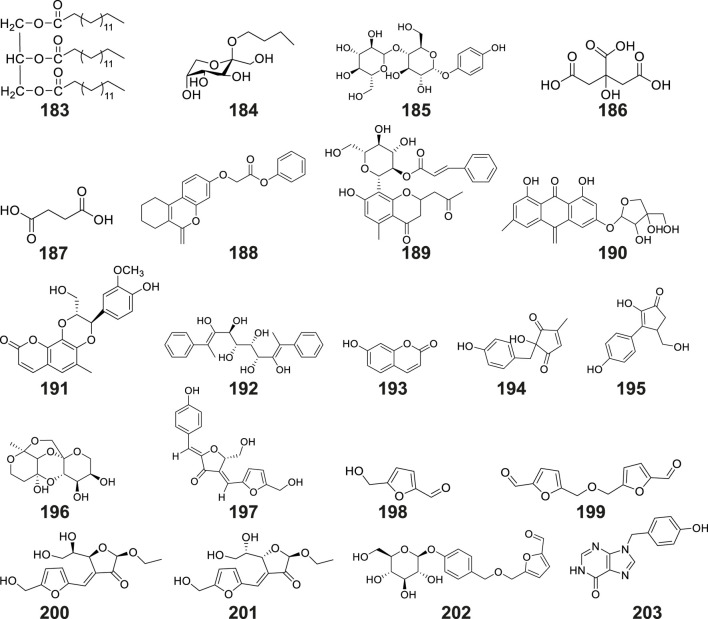
Structures of other compounds isolated and identified from *G. conopsea*.

The tubers of *G. conopsea* were also found to yield a diverse array of other secondary metabolites (Table 2; Figure 10), including: Lipid derivatives: tripalmitin (No. 183) (Li et al., 2008); Saccharides: N-butyl-*β*-D-fructopyranoside (No. 184) (Li et al., 2001) and 4-Hydroxyphenyl-4-O-glucopyranosyl-glucopyranoside (No. 185) (Lin et al., 2021); Organic acids: citric acid (No. 186) and succinic acid (No. 187); Chromene derivatives: benzyl-[(6-oxo-7,8,9,10-tetrahydro-6H-benzo[c]chromen-3yl) oxy]-acetate (No. 188); Anthraquinones: aloeresin A (No. 189) and frangulin B (No. 190); Coumarin-lignan hybrids: cleomiscosin A (No. 191); Cyclic polyols: bis-(methylbenzylidene)-sorbitol (No. 192) (Wang et al., 2020); Phenolic compounds: umbelliferone (No. 193) (Wang et al., 2020), along with two cyclopentenone derivatives: 2-hydroxy-2-(4′-hydroxybenzyl)-4-methylcyclopent-4- ene-1,3-dione (No. 194), and 2-hydroxy-4-hydroxymethyl-3-(4′-hydroxyphenyl) cyclopent-2-enone (No. 195) (Zi et al., 2010); Complex ethers: (2R,3R,4S,5S,7S,8S,9S)-2,3,8,9- tetrahydroxy-7-methyl-pentaoxatetracyclo[6.6.2.0^4,5^.0^7,8^]hexadecane (No. 196); Furan derivatives: (5S)-5-(hydroxymethyl)-4-[(E)-[5′-(hydroxymethyl)furan-2′-yl]methyl ene]-2-[(Z)-(4″-hydroxyphenyl)methylene] tetrahydrofuran-3-one (No. 197), 5-hydroxymethyl-2-furaldehyde (No. 198), Bis - (5-formylfurfuryl) ether (No. 199), pollenfuran A (No. 200), pollenfuran B (No. 201) and 5- ((4-O - β - D-glucopyranosylbenzyloxy)methyl)-furan-2-carboxaldehyde (No. 202) (Qin et al., 2024b); And finally purine analogs: 9-*p*-hydroxybenzylhypoxanthine (No. 203) (Qin et al., 2024a).

### 6.10 Polysaccharides

Beyond its documented small-molecule constituents, *G. conopsea* has been identified as a significant source of bioactive polysaccharides. A comparative study evaluating five extraction methodologies (hot water, enzyme-assisted, ultrasound- assisted, ultrasound-enzyme hybrid, and microwave-assisted) demonstrated that enzyme-assisted and ultrasound-assisted protocols produced polysaccharides with superior anti-oxidant capacity, establishing these as optimal methods for isolating functional polysaccharides from this species (Li F. W. et al., 2021). Structural characterization by Zhang and Borjihan (2005) revealed that the purified polysaccharide from *G. conopsea* tubers was predominantly composed of glucose and mannose in a molar ratio of 1:1.5, with a number average molecular weight Mn = 3.21 × 10^4^, a weight average molecular weight Mw = 8.03 × 10^4^, and a polydispersity of 2.5021. Advanced structural analysis employing ^13^C-NMR and Smith degradation techniques identified (1→3)-linked glycosyl residues as the predominant structural motif, complemented by minor (1→4) linkages. FT-IR and ^1^H-NMR spectral data further confirmed the β-configuration of glycosidic bonds. Recent multi-analytical investigations utilizing HPSEC-MALLS/RID and PACE- based carbohydrate mapping have characterized water-soluble polysaccharides from *G. conopsea* tubers across seven Chinese ecoregions. These studies unveiled a complex glycosidic architecture containing α-1,4- and β-1,3(4)-glucosidic bonds, α-1,5-arabinosidic bonds, β-1,4-mannosidic bonds, and α-1,4-D-galactosidic bonds. Notably, bioactivity assessments demonstrated that nitric oxide production in RAW 264.7 macrophages showed significant correlation with specific structural features: α-1,5-arabinosidic and β-1,3(4)-glucosidic bonds exhibited moderate immunomodulatory effects, while α-1,4-D-galactosiduronic and β-1,4-mannosidic bonds displayed particularly pronounced bioactivity (Lin et al., 2015).

Research indicates that polysaccharides from *G. conopsea* tubers exhibit significant bioactivities, including anti-inflammatory, immunomodulatory, anti-aging, fatigue-alleviating, hypoxia tolerance-enhancing, and anti-radiation effects (Yu, 2017). Their content ranges from 11.07% to 25.05% in crude extracts (Yang, 2010; Yu, 2017; Kong, 2024), establishing polysaccharides as critical functional components of this medicinal plant. However, key structural and mechanistic aspects remain uncharacterized: precise structural features, primary target tissues, and molecular targets of interaction are yet to be elucidated. Intensified research efforts are warranted to advance their development and utilization.

## 7 Pharmacological activities

**FIGURE 11:**
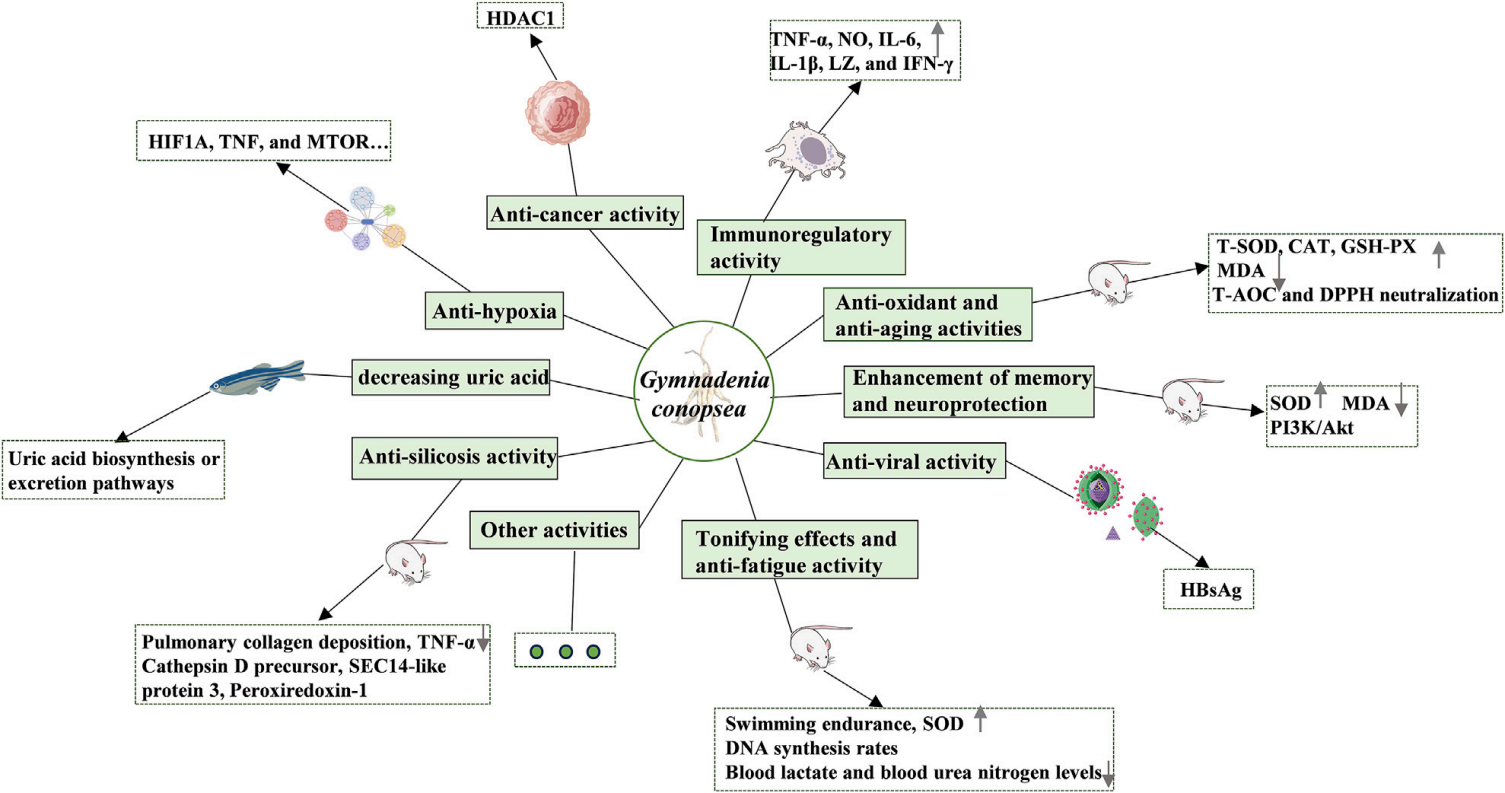
The pharmacological activities of *G. conopsea*.

Modern pharmacological studies have established that *G. conopsea* exhibits a diverse pharmacological profile, encompassing immunomodulation, anti-aging, anti-oxidant activity, neuroprotection, memory enhancement, fatigue resistance, antiviral effects, gastric ulcer mitigation, anti-allergy properties, silicosis inhibition, and sedative-hypnotic functions (Table 3; Figure 11).

**TABLE 3:** Pharmacological effects of *G. conopsea* extracts.

Pharmacological effects	Extract	Model	Dosage and administration	Pharmacological effects	Positive drugs and dosage	Reference
Immunoregulatory activity	The polysaccharides (GC) were prepared through defatting with 80% petroleum ether, removal of ethanol-soluble impurities using 80% ethanol, aqueous extraction, ethanol precipitation (100%), and final deproteinization treatment	RAW264.7 murine macrophages	*In vitro* treatment (0.625, 1.25, 2.5, 5, 10 mg/mL)	GC exert immunomodulatory and anti-inflammatory effects through the regulation of immune adhesion and secretory functions, as well as the inhibition of pyroptosis	10 μg/mL LPS	Kong (2022)
Immunoregulatory activity	The crude polysaccharides (GP3) were prepared through methanol defatting, aqueous extraction, 95% ethanol precipitation, and deproteinization	A model of Kunming strain mice (weighing 18–22 g, with an equal number of males and females) was established by subcutaneous injection of dexamethasone at a dose of 1.25 mg/kg	GP3 was administered orally via gavage at low (50 mg/kg), medium (100 mg/kg), and high (200 mg/kg) doses for 7 consecutive days	GP3 demonstrated the ability to restore immune function in immunosuppressed mice by improving both cellular and humoral immunity	—	Shang et al. (2015)
Immunoregulatory activity	The polysaccharides (CP) were prepared through methanol defatting, aqueous extraction, 95% ethanol precipitation, and deproteinization	Kunming strain mice: weighing 18∼22 g, with an equal number of males and females	CP was administered orally at low (10 μg/g), medium (50 μg/g), and high (100 μg/g) doses daily for 4 consecutive weeks	CP significantly enhanced macrophage phagocytosis and serum lysozyme levels in mice, while promoting delayed-type hypersensitivity responses and moderately elevating thymic and splenic indices	—	Shang et al. (2014)
Anti-oxidant and anti-aging activities	The polysaccharides were prepared through defatting with petroleum ether, removal of impurities using 80% ethanol, aqueous extraction, precipitation with 100% ethanol, and deproteinization	A Kunming mouse model was established by subcutaneous injection of 0.5 g/kg D-galactose solution into the neck and back, with an equal number of male and female mice	The polysaccharides were administered orally at low (0.05 g/kg), medium (0.10 g/kg), and high (0.20 g/kg) doses daily for 60 consecutive days	High-dose polysaccharides enhanced T-SOD, CAT, and GSH-Px activities in serum, cerebral, and hepatic tissues of aging mice, concurrently reducing MDA levels while improving body weight and hepatic index	21.6 mg/kg Vitamin E	Yu et al. (2018)
Anti-oxidant and anti-aging activities	The polysaccharides (GCP) were obtained through aqueous extraction followed by precipitation with 80% ethanol	House-fed male Small-Tailed Han sheep (6 months old, 35.0 ± 4.0 kg) subjected to oxidative stress induced by intraperitoneal injection of Diquat (10 mg/kg body weight)	30 mg/kg of GCP mixed into feed, oral administration via daily feed intake	GCP could improve the growth performance of mutton sheep, alleviate the decline of growth performance and anti-oxidant performance caused by oxidative stress	—	Sa et al. (2020)
Anti-oxidant and anti-aging activities	The polysaccharides were obtained through defatting with 100% ethanol, water extraction, precipitation with 100% ethanol, and removal of protein	Forty-eight female Kunming mice (aged 6–8 weeks, body weight 20–25 g) were exposed to 5.0 Gy^60^Co-γ radiation at a dose rate of 0.8 Gy/min	Low (150 mg/kg), medium (300 mg/kg), and high (600 mg/kg) doses of polysaccharides were administered intragastrically at a volume of 0.5 mL daily for 5 consecutive days postirradiation	The polysaccharides showed therapeutic potential in alleviating the damage to hematopoietic and anti-oxidant functions in mice caused by^60^Co γ-ray irradiation	—	Feng et al. (2022)
Anti-oxidant and anti-aging activities	69% methanol extract of *G. conopsea*	The radical scavenging activity assays for DPPH, ABTS•^+^, H_2_O_2_, ferrous sulfate and salicylic acid-ethanol radical	2, 4, 6, 8, 10 mg/mL	The extract showed dose-dependent antioxidant activities	2, 4, 6, 8, 10 mg/mL VC	Kong (2024)
Anti-oxidant and anti-aging activities	75% and 95% ethanol extracts of *G. conopsea*	The total antioxidant capacity (T-AOC), DPPH, and superoxide anion radical scavenging activity assays	1 g/L	T-AOC and DPPH radical scavenging ability of the 95% ethanol extract were significantly higher than those of the 75% ethanol extract	—	Chen and Liu (2024)
Enhancement of memory and neuroprotection	90% ethanol extract of *G. conopsea*	Aβ25-35 (20 μM) was used to induce PC12 cells to establish an *in vitro* Alzheimer’s disease (AD) cell model	low (2.5 μg/mL), medium (5.0 μg/mL), and high (10.0 μg/mL)	The extract demonstrated significant cytoprotective effects, including attenuation of cellular damage, suppression of apoptosis, facilitation of cellular repair, and alleviation of AD-induced toxicity	—	Cheng (2024b)
Enhancement of memory and neuroprotection	95% ethanol extract of *G. conopsea* (CE)	According to the rat brain stereotaxic atlas of Paxinos and Watson, 1 μL of IBO was injected bilaterally to create a cholinergic lesion model in male SD rats	CE was administered orally at a dosage of 5 mg/kg for 28 consecutive days	CE exhibited neuroprotective effects by enhancing AChE expression and reducing neuronal damage	—	Yu et al. (2013)
Enhancement of memory and neuroprotection	95% ethanol extract of *G. conopsea* (GC) and milk processed *G. conopsea* (MGC)	140 μM AlCl_3_-induced AD zebrafish model	0.1, 0.41, 0.82 mg/mL	All dosages of GC and MGC were capable of effectively reducing brain cell apoptosis and ameliorating the behavioral abnormalities of zebrafish induced by AlCl_3_. The 0.1 mg/mL MGC treatment group outperformed the positive control DPZ	8 μM Donepezil	Yu (2024b)
Enhancement of memory and neuroprotection	Water extract of *G. conopsea*	After feeding with a self-prepared high-fat and high-sugar diet (59% basal diet, 20% sucrose, 18% lard, and 3% egg yolk) for 1 month, a diabetic SD rat model was established by intraperitoneal injection of STZ at a dose of 35 mg/kg	The water extract was administered orally at graded doses (0.6, 1.2 and 2.4 g/kg) for 30 consecutive days	The water extract showed significant neuroprotective, anti-oxidant, and hypoglycemic effects in type 2 diabetic rats with cognitive impairment	0.2 g/(kg·d) Metformin	Shi et al. (2023b)
Enhancement of memory and neuroprotection	Ethanol extract of *G. conopsea*	Male C57BL/6J mice were used to simulate high-altitude hypoxia-induced brain injury. The mice were exposed to a simulated high altitude of 4,000 m in a decompression chamber for 24 h	The extract was administered orally at a dose of 750 mg/kg for 30 consecutive days without positive control	The extract showed neuroprotective effects against hypoxia-mediated brain injury by modulating gene expression and reducing inflammation	—	Zhang et al. (2020)
Sedative and hypnotic activities	The liquid of *G. conopsea* prepared with distilled water	SPF-grade Kunming mice (male and female, 20∼30 g)	High dose (0.2 g/mL, 10 mL/kg), low dose (0.1 g/mL, 10 mL/kg), administered orally, twice a day for 7 consecutive days	*G. conopsea* can inhibit the spontaneous activity of mice, reduce the number of forelimb lifts, and significantly prolong the sleep duration induced by a suprathreshold dose of sodium pentobarbital, as well as increase the number of mice falling asleep induced by a subthreshold dose of sodium pentobarbital	0.004 g/kg Valium tablet	Zhou et al. (2009)
Sedative and hypnotic activities	75% ethanol extract of *G. conopsea*	Kunming mice (both male and female, 20 ± 2 g)	High (4 g/kg), medium (2 g/kg), and low-dose (1 g/kg) extracts were administered orally for 7consecutive days	The ethanol extract of *G. conopsea* significantly reduces the number of spontaneous activities in mice within 5 min, demonstrating notable sedative activity. Moreover, it markedly decreases the number of writhing responses induced by acetic acid in mice, improves the pain threshold, and exhibits significant analgesic effects	4 mg/kg Valium tablet	He et al. (2017)
Tonifying effect	The botanical suspension solution derived from *G. conopsea* processed products utilizing goat milk and cow milk	Kunming mice, (both male and female, 18∼22 g); Wistar rats (both male and female, 180∼220 g)	Respectively administered orally at a dose of 2 g/kg and 2 mL/100 g for 7 consecutive days	The goat milk processed product showed a significant prolonging effect on the swimming time of mice. Both the goat and cow milk processed products showed a significant enhancing effect on the activity of serum SOD in rats, and both have certain nourishing and strengthening effects	—	Jin and Wang (2009)
Tonifying effect	The solution of *G. conopsea* prepared with distilled water	The kidney deficiency model in SPF Kunming male mice was established by intramuscular injection of hydrocortisone at a dose of 25 mg/kg	Two dose groups (high dose: 0.2 g/mL at 10 mL/kg; low dose: 0.1 g/mL at 10 mL/kg) were administered via oral gavage once daily for 10 consecutive days	*G. conopsea* has the effect of tonifying the kidney and strengthening the body in mice with kidney deficiency caused by hydrocortisone	—	Lin (2009)
Anti-fatigue activity	The *G. conopsea* samples were prepared using three different processing methods: goat milk processing, water processing, and 5% *Gardenia jasminoides* solution processing	Kunming mice (both male and female)	Three dose groups (high dose 4 g/kg, medium dose 2 g/kg, and low dose 1 g/kg) were administered via oral gavage once daily for 7 consecutive days	*G. conopsea* exhibited significant anti-fatigue and anti-oxidant activities, and the sheep milk processing method effectively preserves its active pharmacological components	1 g/kg Rhodiola	He (2016)
Anti-fatigue activity	The solution of *G. conopsea* prepared with distilled water	Kunming mice (both male and female)	Three dose groups (high dose 40 g/kg, medium dose 20 g/kg, and low dose 10 g/kg) were administered via oral gavage once daily for 6 consecutive days	*G. conopsea* could obviously prolong the time of swimming to exhaustion under load in mice, demonstrating anti - fatigue effects	20 g/kg Ginseng (Cultivated *Panax ginseng*) Mixture	Zhao and Liu (2011)
Anti-fatigue activity	The crude polysaccharides (GCP) from *G. conopsea* were obtained through aqueous extraction followed by ethanol precipitation	Kunming species male mice	Three dose groups (high dose 0.20 g/kg, medium dose 0.10 g/kg, and low dose 0.05 g/kg) were administered via oral gavage daily for 30 consecutive days	GCP prolonged the swimming time of mice, reduced blood lactic acid levels, and increased liver glycogen content. The high-dose group showed the most significant anti-fatigue effects	0.09 g/kg American Ginseng and Rhodiola Aqueous Solution	Yu (2017)
Anti-viral activity	Water extract of *G. conopsea*	Hepatitis B virus surface antigen (HBsAg) inhibition Model	The water extract was added to the culture medium at concentrations of 0.5 mg/50 μL, 1.5 mg/50 μL, 3 mg/50 μL, and 6 mg/50 μL	The extract demonstrated moderate HBsAg inhibitory efficacy with rapid-acting and time-stable characteristics	—	Lu et al. (2002)
Preventing and treating gastric ulcers	The solution of *G. conopsea* prepared with distilled water	The gastric ulcer model in male SD rats was established by administering 7.5 mL/kg of a hydrochloric acid-ethanol mixture via gavage once daily for 3 consecutive days	Two dose groups were administered via oral gavage once daily for 9 consecutive days: high dose (0.2 g/mL, 10 mL/kg) and low dose (0.1 g/mL, 10 mL/kg)	The solution of *G. conopsea*, particularly at the high dose, demonstrated significant anti-ulcer and ulcer-healing effects	1.95 g/kg Ranitidine	Jiang et al. (2009)
Preventing and treating gastric ulcers	The solution of *G. conopsea* prepared with distilled water	The gastric ulcer model in SD male rats was established by intragastric administration of a hydrochloric acid-ethanol mixture at a dose of 0.75 mL/100 g	Two dose groups were administered orally once daily for 9 consecutive days: high dose (0.2 g/mL, 1 mL/100 g) and low dose (0.1 g/mL, 1 mL/100 g)	*G. conopsea* could inhibit gastric ulcers and reduce the MDA content in both serum and gastric tissue in rats	—	Lin (2009)
Anti-anaphylaxis activity	Methanol extract of *G. conopsea* tubers, and fractions eluted with methanol and water	Inject 10 μL of anti-DNP IgE (20 μg/mL). Then, inject 0.25 mL of PBS containing 2% Evans blue and 0.25 mg of DNP-BSA intravenously to establish a passive allergy model in male ddy mice	Methanol extract: 500, 1,000 mg/kg; methanol-eluted fraction: 100, 200 mg/kg; water-eluted fraction: 1,000 mg/kg	The methanolic extract demonstrated a remarkable antiallergic effect in inhibiting ear passive cutaneous anaphylaxis reactions in mice	100, 200 mg/kg Tranilast	Matsuda et al. (2004)
Anti-silicosis activity	60% ethanol extract of *G. conopsea*	The silicosis model in Wistar male rats was established by intratracheal injection of 1 mL of a 50 g/L sterile silica dust suspension using the bronchial exposure method	A 2 mL volume of *G. conopsea* ethanol extract (10 g/kg body weight) was administered via intragastric gavage daily	The ethanol extract demonstrated inhibitory effects on silica-induced pulmonary fibrosis in rats, concomitant with downregulation of tumor necrosis factor-alpha (TNF-α) expression in pulmonary tissues	—	Zeng et al. (2007)
Anti-silicosis activity	60% ethanol extract of *G. conopsea* (GcAE)	The silicosis model in Wistar male rats was established by intratracheal injection of 1 mL of a 50 g/L sterile silica dust suspension using the bronchial exposure method	GcAE was administered at a dose of 8 g/kg per day (calculated based on the amount of crude drug) via intragastric gavage	GcAE significantly enhances the activities of SOD and GPx, reduces MDA levels and the lung coefficient, and decreases the synthesis of Type I and III collagen in lung tissue	—	Wang et al. (2007)
Anti-silicosis activity	60% ethanol extract of *G. conopsea* (GcEE)	The silicosis model in Wistar male rats was established by intratracheal injection of 1 mL of a 50 g/L sterile silica dust suspension using the bronchial exposure method	GcEE was administered at a dose of 8 g/kg per day (calculated based on the amount of crude drug) via intragastric gavage, Tetrandrine (50 mg/kg/3d) was used as the positive control	GcEE significantly reduces lung coefficient and Types I/III collagen synthesis in silica-exposed rats, with effects comparable to Tetrandrine	50 mg/(kg·3 days) Tetrandrinum	Wang et al. (2008a)
Anti-silicosis activity	60% ethanol extract of *G. conopsea* (GcAE)	The silicosis model in Wistar male rats was established by intratracheal injection of 1 mL of a 50 g/L sterile silica dust suspension using the bronchial exposure method	GcAE was administered at a dose of 8 g/kg per day (calculated based on the amount of crude drug) via intragastric gavage	GcAE can significantly inhibit the overexpression of TNF-a in rat lung tissue induced by silica dust	—	Wang et al. (2008b)
Anti-silicosis activity	60% ethanol extract of *G. conopsea* (GcAE)	The silicosis model in SPF-grade inbred male Wistar rats was established by intratracheal injection of 1 mL of a 50 mg/mL sterile silica dust suspension using the tracheal exposure method	GcAE was administered at a dose of 8 g/kg body weight per day via intragastric gavage	GcAE demonstrated significant anti-fibrotic, anti-inflammatory, and anti-oxidant effects in a rat model of early-stage silicosis	50 mg/(kg·3 days) Tetrandrine	Chen (2008)
Anti-silicosis activity	60% ethanol extract of *G. conopsea* (GcAE)	The silicosis model in SPF-grade inbred male Wistar rats was established by intratracheal injection of 1 mL of a 50 mg/mL sterile silica dust suspension using the tracheal exposure method	GcAE was administered at a dose of 8 g/kg body weight per day via oral gavage	GcAE showed multi-faceted pharmacological effects, including anti-fibrotic, anti-inflammatory, anti-oxidant, and anti-apoptotic properties	—	Chen (2009)
Anti-silicosis activity	60% ethanol extract of *G. conopsea* (GcAE)	The silicosis model in clean-grade Wistar male rats was established by intratracheal injection of 1 mL of a 50 mg/mL sterile silica dust suspension using the tracheal exposure method	GcAE was administered at a dose of 8 g/kg body weight per day via oral gavage	GcAE exhibited effects similar to the positive control drug Tetrandrine, demonstrating the potential to alleviate inflammation and fibrosis induced by silica dust stimulation during the early and middle stages	50 mg/(kg·3 days) Tetrandra	Wu et al. (2010)
Lowering blood lipid and protecting the liver	70% ethanol extract of *G. conopsea*	The hyperlipidemia model in SPF-grade SD rats was established by feeding a high-fat diet consisting of 78.8% basal feed, 10% lard, 10% egg yolk powder, 1% cholesterol, and 0.2% bile salts	Three dose groups (high dose 5 g/kg, medium dose 2.5 g/kg, and low dose 1.25 g/kg) were administered via oral gavage once daily for 14 consecutive days	The extract effectively reduced lipid levels in both serum and liver tissue, and it protected against liver injury induced by hyperlipidemia	3 mg/kg Lovastatin	Zhang et al. (2013)
decreasing uric acid	75% and 95% ethanol extracts of *G. conopsea*	The combination of PO and xanthine sodium salt (XSS) was used to establish an XOD model in zebrafish	Zebrafish embryos were treated with the extracts at different concentrations for 24 h	Both extracts significantly reduced uric acid levels in hyperuricemia zebrafish, with the 95% extract showing a more pronounced effect	—	Chen et al. (2022b)
Anti-hypoxia	Twenty active ingredients of *G. conopsea*	Network pharmacology analysis	—	*G. conopsea* achieves the process of anti-hypoxia through multiple components and multiple targets. The main active components play an anti-hypoxic role by acting on targets such as HIF1A, TNF, and MTOR	—	Liang et al. (2021)

### 7.1 Immunoregulatory activity

As prominent immunomodulators, plant polysaccharides play a critical role in immune system regulation. *G. conopsea*, containing up to 25.05% polysaccharides (Kong, 2024), serves as a key reservoir of bioactive metabolites. Accumulating evidence indicates that its polysaccharides significantly enhance immune cell functionality—including macrophages and lymphocytes—thereby amplifying host immunity (Yang et al., 2021)^.^ Kong (2022) employed an optimized water extraction-alcohol precipitation protocol to isolate *G. conopsea* polysaccharides. Using RAW264.7 macrophages as an *in vitro* model, the study demonstrated that these polysaccharides not only upregulated macrophage proliferation and phagocytosis but also activated Fc/C3b receptors and stimulated the secretion of TNF-α, NO, IL-6, IL-1β, LZ, and IFN-γ. Notably, under LPS stimulation, the polysaccharides paradoxically suppressed macrophage phagocytic activity and cytokine release, revealing a bidirectional immunoregulatory mechanism. Complementing these findings, Shang et al. (2015) prepared crude *G. conopsea* polysaccharides through methanol degreasing, water extraction, and 95% alcohol precipitation. In dexamethasone-induced immunosuppressed mice, medium (100 mg/kg) and high (200 mg/kg) doses of *G. conopsea* polysaccharides markedly elevated thymic/splenic indices and macrophage phagocytic capacity, confirming dose-dependent immunomodulatory effects. Shang et al. (2014) further reported that *G. conopsea* polysaccharides augmented peritoneal macrophage activity, increased serum lysozyme levels, promoted delayed-type hypersensitivity, and normalized immune organ weights, suggesting broad-spectrum immunoregulation.

Compared to the extensive research on the *in vivo* immunomodulatory effects of polysaccharides, studies on their pharmacokinetic profiles, primary target organs, membrane-bound receptors, and downstream signaling pathways remain limited. Additionally, the modulation of gut microbiota—a research hotspot in recent years—is closely linked to systemic immunity (Wang et al., 2023). It is thus imperative to systematically investigate whether *G. conopsea* polysaccharides exert immunomodulatory effects via gut microbiota regulation in whole-animal models.

### 7.2 Anti-oxidant and anti-aging activities

Beyond immunomodulation, *G. conopsea* polysaccharides exhibit potent anti-oxidant and anti-aging properties. A polysaccharide fraction (89.80% purity) obtained through petroleum ether degreasing, ethanol impurity removal, water extraction, and alcohol precipitation demonstrated dose-responsive protective effects in D-galactose-induced aging mice. High-dose administration (0.20 g/kg) over 60 days significantly upregulated T-SOD, CAT, and GSH-PX activities in serum, brain, and liver tissues while suppressing MDA levels—effects attributed to enhanced anti-oxidant enzyme activity and reduced lipid peroxidation (Yu et al., 2018). Sa et al. (2020) isolated a 12.16% pure polysaccharide fraction utilizing an optimized aqueous extraction protocol followed by alcohol precipitation (Yu, 2017) and evaluated its efficacy in Diquat-challenged Small-tailed Han sheep. Dietary supplementation for 15 days elevated SOD/GSH-Px levels and reduced MDA concentrations, effectively counteracting oxidative stress-induced metabolic impairments. Radiation protection studies revealed that polysaccharide administration (150–600 mg/kg) post 60Co-γ irradiation dose-dependently restored hematopoietic function, amplified anti-oxidant defenses, and accelerated repair of radiation-induced damage (Feng et al., 2022).

Small-molecule extracts also contribute to anti-oxidant activity. Dose-dependent scavenging of DPPH, ABTS^+^, and hydroxyl radicals was observed in 69% methanol extracts from 14 plant batches (Kong, 2024). Comparative analysis by Chen and Liu (2024) showed that 95% ethanol extracts possessed superior total anti-oxidant capacity (T-AOC) and DPPH neutralization compared to 75% extracts, though with reduced superoxide anion scavenging efficiency. Similarly, Study by Morikawa et al. (2006) confirmed that the methanol extract of *G. conopsea* possesses scavenging activity against DPPH and superoxide anion (O_2_^−^) radicals. Its major active compounds were enriched in the methanol and acetone eluates from Diaion HP-20 column chromatography, leading to the isolation and identification of 11 compounds with significant activity (see Table 4). Among these, the compound blestriarene A (No. 100) demonstrated potent activity, with SC_50_ of 5.8 μM for DPPH scavenging, IC_50_ of 0.27 μM against formazan formation, and 4.5 μM for xanthine oxidase inhibition, outperforming the positive controls α-Tocopherol and (+)-Catechin. Furthermore, at the cellular level, the compound dactylorhin B (No. 3) was reported to alleviate β-amyloid_23-35_-induced mitochondrial damage and reduce apoptosis in SH-SY5Y cells by inhibiting reactive oxygen species (ROS) (Zhang et al., 2006). Importantly, the principal bioactive component gastrodin (No.30) has been pharmacologically validated as a potent anti-oxidant and anti-aging agent (Shang et al., 2024). Collectively, these findings underscore the robust anti-oxidant and anti-aging activities of *G. conopsea*.

**TABLE 4:** Pharmacological effects of compounds purified from the tubers of *G. conopsea*.

Pharmacological effects	Compounds	Model	Effects	Reference
Anti-oxidant activity	gymconopin A (No. 93)	DPPH, Formozan formation, Xanthine oxidase	^a^SC_50_ 29.2 μM, ^b^IC_50_ 45.8 μM, ^c^IC_50_ > 100 μM	Morikawa et al. (2006)
	gymconopin B (No. 94)		^a^SC_50_ 33.4 μM, ^b^IC_50_ 21.5 μM, ^c^IC_50_ > 100 μM	
	2-Methoxy-9,10-dihydrophenanthrene-4,5-diol (No. 98)		^a^SC_50_ 31.2 μM, ^b^IC_50_ > 100 μM, ^c^IC_50_ no	
	4-Methoxy-9,10-dihydrophenanthrene-2,7-diol (No. 99)		^a^SC_50_ 12.7 μM, ^b^IC_50_ 0.95 μM, ^c^IC_50_ 44.0 μM	
	1-(4-Hydroxybenzyl)-4-methoxy-9,10-dihydrophenanthrene-2,7-diol (No. 96)		^a^SC_50_ 8.2 μM, ^b^IC_50_ 0.19 μM, ^c^IC_50_ 30.5 μM	
	1-(4-Hydroxybenzyl)-4-methoxyphenanthrene-2,7-diol (No. 97)		^a^SC_50_ 15.7 μM, ^b^IC_50_ 9.4 μM, ^c^IC_50_ > 100 μM	
	blestriarene A (No. 100)		^a^SC_50_ 5.8 μM, ^b^IC_50_ 0.27 μM, ^c^IC_50_ 4.5 μM	
	batatacin III (No. 76)		^a^SC_50_ > 40 μM, ^b^IC_50_ 82.8 μM, ^c^IC_50_ > 100 μM	
	3′,5-Dihydroxy-2-(4-hydroxybenzyl)-3-methoxybibenzyl (No. 78)		^a^SC_50_ > 40 μM, ^b^IC_50_ 9.3 μM, ^c^IC_50_ 72.9 μM	
	3,3′-Dihydroxy-2-(4-hydroxybenzyl)-5-methoxybibenzyl (No. 79)		^a^SC_50_ > 40 μM, ^b^IC_50_ 13.4 μM, ^c^IC_50_ 45.1 μM	
	3,3′-Dihydroxy-2,6-bis(4-hydroxybenzyl)-5-methoxybibenzyl (No. 81)		^a^SC_50_ > 40 μM, ^b^IC_50_ 13.4 μM, ^c^IC_50_ 65.2 μM	
	α-Tocopherol^e^		^a^SC_50_ 11.0 μM, ^b^IC_50_ no, ^c^IC_50_ no	
	(+)-Catechin^e^		^a^SC_50_ 6.0 μM, ^b^IC_50_ 1.5 μM, ^c^IC_50_ > 10 μM	
	arctigenin (No. 154)	Fe^+2^-cystine induced rat liver microsomal lipid peroxidation	Inhibitory rate 53% at 1 μM	Yue et al. (2010)
	lappaol A (No. 155)		Inhibitory rate 59% at 1 μM	
	lappaol F (No. 156)		Inhibitory rate 52% at 1 μM	
	vitamin E^e^		Inhibitory rate 35%^f^	
	dactylorhin B (No. 3)	β-amyloid _25–35_ (50 μM) induced ROS burst in SH-SY5Y cells	Inhibition rate was approximately 50% at 10 μM	Zhang et al. (2006)
Anti-HIV activity	(−)-4-[β-D-glucopyranosyl-(1→4)-β-D-glucopyranosyloxy]benzyl alcohol (No. 28)	VSVC/HIV-luc model in 293 cell lines	Inhibitory rate 9.0% at 10 μM	Zi et al. (2008a)
	(−)-4-[β- D-glucopyranosyl-(1→3)-β-D-glucopyranosyloxy] benzyl ethyl ether (No. 31)		Inhibitory rate 5.0% at 10 μM	
	(−)-(2R,3S)-1-[4-β-D-glucopyranosyloxybenzyl]-4-methyl-2-isobutyltartrate (No. 26)		Inhibitory rate 6.2% at 10 μM	
	Cyclo[gly-L-S-(4-hydroxybenzyl)]cys (No. 139)		Inhibitory rate 11.9% at 10 μM	
	2-Hydroxy-2-(4′-hydroxyphenylmethyl)-4- methylcyclopent-4-en-1,3-dione (No. 149)		Inhibitory rate 11.3% at 10 μM	
	2-Hydroxy-3-(4-hydroxyphenyl)-4-hydroxymethylcyclopent-2-enone (No. 195)		Inhibitory rate 0.6% at 10 μM	
	coelovirins E (No. 8)		Inhibitory rate 13.3% at 10 μM	
	dactylorhin E (No. 9)		Inhibitory rate 5.1% at 10 μM	
	dactylorhin B (No. 3)		Inhibitory rate 10.0% at 10 μM	
	militarine (No. 2)		Inhibitory rate 2.4% at 10 μM	
	gastrodin (No. 30)		Inhibitory rate 0.6% at 10 μM	
	zidovudine^e^		Inhibitory rate 85.6% at 100 nM	
	lamivudine^e^		Inhibitory rate 47.4% at 10 nM	
Neuroprotection	dactylorhin B (No. 3)	Damage of SH-SY5Y cells and rat brain mitochondrial function induced by β-amyloid _25–35_	^d^EC_50_ = 7.0 μM Attenuated β-amyloid _25–35_-induced mitochondrial damage	Zhang et al. (2006)
	No. 22-31	*In vitro* assays for acetylcholine esterase and monoamine oxidase inhibitory activities	All were inactive at 10 μM	Zi et al. (2008a)
	donepezil^e^		Inhibitory rate 77.2% at 10 μM	
	pargyline^e^		Inhibitory rate 94.5% at 10 μM	
	gymnaoxa (No.196)	Oxygen–glucose deprivation/reoxygenation (OGD/R)-injured PC12 cells injury model	12.5–50 μM active	Qin et al. (2024b)
	gymnafuran B (No. 197)		1.06–3.12 μM active	
	bis (5-formylfurfuryl) ether (No. 199)		25–50 μM active	
	5-((4-O-β-D-glucopyranosylbenzyloxy)methyl)-furan-2-carbaldehyde (No. 202)		12.5–25 μM active	
	edaravone^e^		12.5–50 μM active	
	No. 54/71/73/74/75/203		All tested compounds exhibited neuroprotective effects to varying degrees at concentrations of 12.5, 25, and 50 μM. compound No. 75 was comparable to that of the positive control edaravone at the same concentrations	Qin et al. (2024a)

Note:

^a^ Concentration required for 50% reduction of 40 mM DPPH, radical.

^b^ Concentration required for 50% reduction of Formozan formation.

^c^ Concentration required for 50% reduction of Formozan formation.

^d^ Concentration required for 50% reduction of β-amyloid_25-35_ induced cytotoxicity; “no” Indicates the IC_50_ value that was not measured.

^e^ Represented as the positive control compound.

^f^ Indicates that the specific treatment concentration is not specified.

### 7.3 Enhancement of memory and neuroprotection

*G. conopsea* has demonstrated significant therapeutic potential in neurodegenerative disorders, particularly for Alzheimer’s disease (AD) prophylaxis and cognitive enhancement (Luo, 2021; Guo et al., 2022). Mechanistic studies revealed that a 90% ethanol refluxed extract effectively attenuated β-amyloid_25-35_(Aβ_25-35_, 20 μM)-induced apoptosis in PC12 cell model (Cheng, 2024b). Similarly, a 95% ethanol extract alleviated aluminum trichloride-induced behavioral abnormalities in zebrafish by inhibiting neuronal apoptosis (Yu, 2024b), providing preliminary evidence for its anti-AD efficacy. Given the critical role of cholinergic neuron degeneration in cognitive decline, Yu et al. (2013) employed an ibotenic acid-induced cholinergic injury model. They demonstrated that 28-day administration of a 95% ethanol extract (5 mg/kg) significantly ameliorated neuropathological changes and upregulated acetylcholinesterase expression. Ina metabolic-related neural damage model, Shi et al. (2023b) observed that an aqueous extract (0.6–2.4 g/kg) reversed high-fat- diet-induced cognitive deficits in diabetic rats. This effect was mediated by reducing fasting blood glucose, increasing superoxide dismutase (SOD) activity, decreasing malondialdehyde (MDA) levels, and elevating PI3K/Akt protein expression levels. Notably,an ethanol extract (750 mg/kg) exhibited neuroprotective effects against hypoxia by downregulating lipocalin-2 (LCN2) protein expression (Zhang et al., 2020; Afridi et al., 2024). However, most of these studies did not characterize the extracts (e.g., via HPLC) or discuss potential active constituents. Nevertheless, they provide a pharmacological foundation for investigating the neuroprotective components within *G. conopsea*.

Focusing on specific neuroprotective compounds, Zhang et al. (2006) found that dactylorhin B (No. 3) effectively mitigated SH-SY5Y cell damage induced by Aβ_25-35_. This protection was achieved by inhibiting reactive oxygen species (ROS) burst and reducing mitochondrial damage. Furthermore, Qin et al. (2024b) reported that compounds NO. 54, 71, 73, 74, 75, 196, 197, 199, 202, and 203 exhibited varying degrees of protective activity in PC12 cells subjected to oxygen-glucose deprivation/reperfusion (OGD/R) injury. Especially, compound (2S)-2-(β-D-pyran-glucosyloxy)-2-(2-methylpropyl)butanedioic acid 4-methyl ester (No. 75) has a significant neuroprotective effect, and its activity is comparable to that of the positive drug edaravone. Further verification through transcriptome, bioinformatics and qPCR suggests that compound No. 75 may exert its protective effect by regulating COL27A1 (Qin et al., 2024a). These findings highlight the potential of these compounds for drug development and establish a solid foundation for elucidating the material basis and underlying molecular mechanisms of *G. conopsea*’s neuroprotective effects. Notwithstanding, the *in vivo* neuroprotective effects of these monomeric compounds, along with their *in vivo* absorption profiles and tissue distribution patterns, particularly the distribution in brain tissue, necessitate further systematic investigations.

### 7.4 Sedative and hypnotic activities

In the pharmacological compendia of Tibetan and Mongolian medicinal traditions, *G. conopsea* is documented for its sedative and hypnotic properties. Research indicates that a solution of *G. conopsea*, prepared with distilled water, demonstrates efficacy in both high-dose (1 mL of solution containing 0.2 g of crude drug, equivalent to a concentration of 0.2 g/mL) and low-dose (1 mL of solution containing 0.1 g of crude drug, equivalent to a concentration of 0.1 g/mL) formulations, in suppressing the spontaneous activity of mice, diminishing the frequency of vertical forelimb lifts, and significantly extending the duration of sleep induced by suprathreshold doses of pentobarbital sodium. Concurrently, it enhances the incidence of mice succumbing to sleep induced by subthreshold doses of pentobarbital sodium, thereby affirming the sedative and hypnotic effects of *G. conopsea* (Zhou et al., 2009). Moreover, a study conducted by He et al. (2017) assessed the sedative efficacy of three doses (1, 2, and 4 g/kg) of a 75% ethanol extract of *G. conopsea* using mouse sedation trials. The findings revealed that the suppression rates of spontaneous mouse activities were 22.5%, 44.9%, and 51.2% respectively, signifying that the 75% ethanol extract of *G. conopsea* exhibits a dose-dependent inhibition of spontaneous mouse activities and possesses notable sedative effects. Although *in vivo* animal models are effective for evaluating and replicating the clinical efficacy of drugs, they are not the optimal choice for identifying bioactive components and elucidating underlying mechanisms. Existing studies have confirmed the presence of sedative compounds in the tubers of *G. conopsea*. Consequently, it is necessary to employ well-established *in vitro* cellular models and molecular docking approaches to further isolate and validate specific bioactive constituents, as well as to clarify their potential molecular mechanisms.

### 7.5 Tonifying effects and anti-fatigue activity

*G. conopsea* is widely recognized in traditional Chinese medicine for its dual tonifying and anti-fatigue properties, targeting conditions such as physical debility, pulmonary and renal insufficiency, emaciation, and fatigue-associated syndromes. As a restorative tonic, it demonstrates efficacy in alleviating cough, asthma, and wasting disorders while enhancing Yang energy consolidation. Modern pharmacological studies validate its role in improving energy metabolism and mitigating fatigue through structured experimental models.

The tubers of *G. conopsea* were powdered and dissolved directly in distilled water to prepare the “*G. conopsea* solution”. Compared to the control group, administration of this solution at high (40 g/kg), medium (20 g/kg), and low (10 g/kg) doses significantly prolonged weight-loaded swimming time in mice, accompanied by increased activity levels, food intake, and mental alertness (Zhao and Liu, 2011). In a hydrocortisone-induced mouse model of kidney yang deficiency, administration of *G. conopsea* solutions (0.2 g/mL and 0.1 g/mL concentration, dosed at 10 mL/kg) significantly improved body weight, kidney coefficient, and DNA synthesis rates in renal and splenic tissues, highlighting its kidney-invigorating and body-strengthening effects (Lin, 2009). Medicinal processing (Paozhi) is a distinctive feature of traditional Chinese medicine preparation, serving purposes such as toxicity reduction and efficacy enhancement. Studies have found that *G. conopsea* processed with goat or cow milk significantly prolongs swimming endurance in mice and elevates serum superoxide dismutase (SOD) activity in rats, with superior effects compared to the crude drug group (Jin and Wang, 2009). Anti-fatigue and hypoxia tolerance tests in mice further confirmed its tonic and invigorating properties (He, 2016). Administration of differently processed *G. conopsea* preparations (goat milk processing, water processing, and processing with 5% *Gardenia jasminoides* solution) at various doses (1, 2, and 4 g/kg) all induced dose-dependent increases in weight-loaded swimming time and grid-hanging duration; the goat milk-processed preparation demonstrated the most potent effects.

Studies by Zhao and Liu (2011) and Lin (2009) provided preliminary evidence for the tonic and invigorating effects of the crude *G. conopsea* drug. Research by Jin and Wang (2009) and He (2016) suggested that milk processing enhances its restorative efficacy. However, some methodological descriptions lack detail: for instance, Jin and Wang (2009). did not specify whether the material was powdered/sieved or the solvent used for suspension; He (2016) did not include a crude drug control group. Furthermore, none of these studies characterized potential bioactive content, HPLC compound profiles, or chemical markers, hindering comparative analysis in subsequent research.

Precise characterization of bioactive metabolites is crucial for promoting the standardization and quality improvement of medicinal plant materials. Research by Yu (2017) identified polysaccharides as the primary bioactive components. Prolonged administration (30 days) of optimally prepared crude *G. conopsea* polysaccharides (0.05, 0.1, and 0.2 g/kg) not only extended weight-loaded swimming time in mice but also reduced blood lactate and blood urea nitrogen levels while increasing hepatic glycogen reserves, indicating dual mechanisms of energy conservation and metabolic waste clearance. This study confirms polysaccharides as key constituents responsible for the tonic and invigorating effects of *G. conopsea*. Beyond polysaccharides, further systematic investigation is warranted to determine whether other small-molecule metabolites in *G. conopsea*—such as benzylester glucosides, stilbenes, and phenanthrenes—contribute to the aforementioned efficacy, and whether synergistic interactions exist between polysaccharides and small molecules.

### 7.6 Anti-viral activity

In traditional medicine, *G. conopsea* has been historically employed in managing chronic hepatitis B (Shang et al., 2017). Lu et al. (2002) evaluated the anti-HBV activity of the aqueous extract of *G. conopsea*. The results showed that the aqueous extract of *G. conopsea* exhibited moderate inhibition of HBsAg, and higher doses led to more significant inhibition. Moreover, this inhibitory effect was rapid and remained stable over time. Complementary studies further identified antiviral properties in the plant’s methanol extract, which suppressed viral polymerase activity (Kimura, 2003). Moreover, Zi et al. (2008a) evaluated the anti-HIV activity of 11 compounds isolated from *G. conopsea* tubers using a VSV-G pseudotyped HIV-luc reporter assay in 293 cells. However, these compounds showed only weak activity (0.6%–13.3% inhibition at 10 μM, see Table 4), significantly lower than the positive control drug. Clearly, research on the antiviral properties of *G. conopsea* remains insufficiently systematic and in-depth. Experience from COVID-19 treatment indicates that while botanical metabolites often exhibit limited direct antiviral potency, they can effectively alleviate symptoms through synergistic mechanisms. These include immune modulation, prevention of cytokine storms, and mitigation of tissue damage (Huang et al., 2021). Therefore, future research should prioritize comprehensive evaluation of *G. conopsea*’s antiviral activity in whole animal models, coupled with *in vitro* models targeting specific mechanisms, to enable precise identification of the active metabolites.

### 7.7 Preventing and treating gastric ulcers

Jiang et al. (2009) established a standardized gastric ulcer model in Sprague-Dawley rats via intragastric administration of hydrochloric acid-ethanol solution (7.5 mL/kg). Their findings revealed that *G. conopsea* significantly attenuated inflammatory responses in ulcerative lesions by modulating capillary permeability and promoting granulation tissue proliferation. These combined effects enhanced gastric mucosal repair capacity and accelerated ulcer healing, with particularly notable efficacy in acute gastric ulcer management. In another parallel study utilizing the same ulcerogenic protocol (7.5 mL/kg HCl-ethanol), researchers further demonstrated that *G. conopsea* exerted dual protective effects: it markedly reduced gastric ulcer index scores and suppressed MDA levels, a key biomarker of oxidative stress. These results corroborate the botanical drug’s robust gastroprotective properties, highlighting its potential in both preventing ulcerogenesis and facilitating mucosal recovery (Lin, 2009). *G. conopsea* whole-component extract demonstrates significant preventive, protective, and reparative effects against gastric ulcers, exhibiting efficacy comparable even to the positive control drug ranitidine (Jiang et al., 2009). This highlights its considerable development potential. However, it is evident that related research reports are limited, and the studies lack depth and systematic rigor. Crucially, the plant material used was not characterized, and no investigation into potential active metabolites was conducted.

### 7.8 Anti-silicosis activity

Silicosis, a progressive pneumoconiosis characterized by bilateral nodular pulmonary fibrosis, arises from chronic inhalation of crystalline silica (SiO_2_) particles. Pharmacological studies in silica-exposed rat models demonstrate that the 60% ethanolic extract of *G. conopsea* (GcAE) effectively mitigates pulmonary fibrosis by upregulating antioxidant enzyme (SOD, GPx) activity, reducing lipid peroxidation product (MDA) levels and lung index, and decreasing Type I/III collagen deposition in lung tissue (Wang et al., 2007; Wang et al., 2008a). GcAE also significantly downregulates TNF-α expression in lung tissue, thereby inhibiting TNF-α-mediated inflammatory cascades and reducing fibrosis (Zeng et al., 2007; Wang et al., 2008b). To further investigate GcAE’s anti-silicosis mechanisms, Chen (2008) employed gene microarray technology to analyze differentially expressed genes in lung tissue. Their data indicate that GcAE counteracts pulmonary fibrosis via multi-target mechanisms, including alleviating oxidative stress, protecting vascular endothelium, and inhibiting lymphocyte-endothelial cell adhesion. Subsequently, Chen (2009) conducted proteomic analysis of differentially expressed proteins in silicotic rat lungs. Results revealed that GcAE intervention significantly upregulated SEC14-like protein 3 (involved in lipid signaling) while downregulating procathepsin D (lysosomal protease regulation) and peroxiredoxin 1 (redox homeostasis), potentially alleviating silica-induced fibrosis by enhancing pulmonary antioxidant defenses and anti-apoptotic capacity. Notably, a comparative pharmacodynamics study by Wu et al. (2010) showed that the 70% ethanolic extract of *G. conopsea* exhibits anti-fibrotic efficacy comparable to the clinical reference drug tetrandrine in silica-challenged models, highlighting its potential as a phytotherapeutic alternative for silicosis treatment.

However, the aforementioned studies exhibit notable limitations requiring future refinement: (1) Limited dose-response assessment: Most utilized only a single dose, leaving dose-dependent effects unconfirmed; (2) Insufficient extract characterization: Lack of qualitative/quantitative profiling of bioactive constituents; (3) Inconsistent positive controls: Absence of reference drug groups in some studies (see Table 4); (4) Inadequate mechanistic validation: Superficial pathway analysis without functional validation of targets.

### 7.9 Other activities

Beyond its primary applications, *G. conopsea* exhibits multifaceted pharmacological activities, including lipid-lowering and hepatoprotective effects, as evidenced by studies demonstrating that 70% ethanolic extracts significantly alleviate hyperlipidemia-induced hepatic damage in rat models through the regulation of lipid metabolism (Zhang et al., 2013). The plant also demonstrates notable hypouricemic properties, likely mediated by its diverse phytochemical metabolites such as flavonoids, polyphenols, alkaloids, terpenes, and phenylpropanoids, which may interfere with uric acid biosynthesis or excretion pathways (Chen T. et al., 2022). Concurrently, network pharmacology analyses by Liang et al. (2021) propose that its bioactive components combat hypoxia via multi-target modulation of hypoxia-inducible factor 1α, TNF, and mTOR (mechanistic target of rapamycin) signaling axes. In anticancer research, conopsamide A (No. 141), a unique ureido-substituted amino acid isolated from tuber ethanol extracts, has emerged as a potential HDAC1 (histone deacetylase 1) inhibitor with epigenetic regulatory capabilities (Lin et al., 2017). Additionally, methanol extracts of the tubers exhibit tranilast-comparable anti-allergic activity, particularly in fractions purified by reverse-phase silica gel chromatography, though the precise bioactive molecules remain to be fully characterized (Matsuda et al., 2004).

## 8 Toxicity

Toxicity is closely associated with the safety of drug administration, representing a pivotal element in pharmaceutical research. In acute oral toxicity tests, BALB/C mice (17–20 g) and SD rats (180–220 g) were administered Wangla (prepared from *G. conopsea* tuber) at doses of 1.00, 2.15, 4.64, and 10.00 g/kg body weight. No mortality or abnormal symptoms occurred, with LD_50_ values exceeding 10.00 g/kg for all groups, classifying the substance as practically non-toxic. Genotoxicity assessments—including mouse bone marrow micronucleus assays (BALB/C mice, 25–30 g body weight, dosage at 1.25, 2.50, 5.00 g/kg) and sperm abnormality tests (BALB/C mice, 25–35 g body weight, dosage at 1.25, 2.50, 5.00 g/kg)—showed no significant increases in micronucleated polychromatic erythrocytes or sperm abnormalities (*P* > 0.05), contrasting sharply with positive controls (cyclophosphamide at 40 mg/kg and 30 mg/kg, respectively; *P* < 0.01). In a 30-day subchronic study, SD rats (95.3 ± 9.4 g) received dietary doses of 1.67, 3.33, and 6.67 g/kg (50–200× human intake). No adverse effects were observed on body weight, food utilization, hematology (hemoglobin, RBC/WBC counts), blood biochemistry (ALT, AST, BUN, creatinine, lipids, glucose, proteins), or organ coefficients (liver, kidney, spleen, gonads), except for isolated focal hepatic necrosis in 5/10 male rats at the highest dose (6.67 g/kg). This finding was deemed spontaneous due to the absence of dose dependency, corroborated histopathologically by normal kidney, stomach, and duodenal tissues. Collectively, the data support the safety of Wangla for oral use as both a medicinal agent and food supplement (Bai and Zheng, 2007).

In another study, He et al. (2023b) conducted a 90-day long-term toxicity study, SPF SD rats (n = 120, 180–200 g, equal sex distribution) were administered *G. conopsea* via daily gavage and medicated feed at three doses: 5.1 g/kg (10×), 10.2 g/kg (20×), and 15.4 g/kg (30× clinical human equivalent dose), followed by a 15-day recovery period. Throughout the experiment, no mortality or behavioral abnormalities (e.g., secretions, altered feces/urine) occurred, and body weight/food intake remained unchanged versus controls. Hematologically, transient elevation of MPV (mean platelet volume) was observed in the 20× and 30× groups at day 45 (*P* < 0.01), but all values normalized by day 90 and recovery. Biochemical analysis revealed significantly decreased creatinine (Crea) and total protein (TP) in the 20× and 30× groups at day 90 (*P* < 0.01), which reversed post-recovery; however, cholesterol (CHO) reduction persisted in the 20× group during recovery (*P* < 0.05). No alterations occurred in ALT, AST, glucose, triglycerides, or other metabolic markers. Critically, organ coefficients (liver, heart, kidneys, spleen, gonads, etc.) showed no differences from controls (*P* ≥ 0.05), and histopathology of 12 organs (including liver, kidneys, heart, lungs, and reproductive tissues) confirmed absence of lesions at all timepoints (45 days, 90 days, 105 days). The study employed no positive toxin controls, as its design focused exclusively on dose-dependent toxicity assessment. These results indicate no evidence of cumulative organ damage or irreversible toxicity, supporting the safety of long-term clinical use; the isolated CHO decrease warrants further investigation but lacks pathological correlation.

The findings of acute and long - term toxicity experiments indicate that *G. conopsea* exhibits no toxicity, which is in line with the outcomes of long - term clinical applications. Its functions, including tonifying effects and neuroprotective properties, endow it with broad prospects for application in the development of future pharmaceuticals and health products.

## 9 Breeding research

Current research on *G. conopsea* cultivation technology remains in its nascent stage, with limited progress in scaling artificial propagation systems. Existing efforts primarily focus on foundational techniques including symbiotic fungal isolation, seed germination optimization, and callus induction protocols. Systematic evaluation of these methodologies is critical for establishing scalable cultivation frameworks to support industrial applications.

### 9.1 Introduction and domestication of wild resources

Wild resource domestication has emerged as a strategic approach to address medicinal resource scarcity and enable artificial cultivation transitions. However, few studies specifically address *G. conopsea* domestication dynamics. Pioneering work by Song et al. (2011) demonstrated that successful transplantation requires microenvironmental fidelity to native habitats, recommending wild plants transfer with intact soil into shaded shelters, coupled with stringent hydration, nutrient, and pest management. Their parallel attempts at seed propagation via direct sowing or sand-burial methods proved unsuccessful due to orchidaceous seed underdevelopment, highlighting reproductive challenges.

Vegetative propagation techniques show partial success: autumn-harvested tubers with multiple buds can undergo ramet division when each segment retains nascent buds, achieving medicinal harvest readiness after 3–5 years (Song et al., 2011). Spring equinox division protocols involve sectioning rhizomes into 1–2 bud-eye segments with retained fibrous roots, followed by plant ash treatment and furrow planting (30 cm row spacing, 8–10 cm plant spacing, 5–6 cm depth), achieving full emergence within 15 days under moisture-controlled conditions (Bao et al., 2024). The research conducted by Bao (2023) revealed that transplanted *G. conopsea* tends to enter a state of dormancy at an earlier stage. The researchers posited that this phenomenon might be attributable to the diminished soil nutrient quality in the transplantation site compared to the native habitat soil of *G. conopsea*. Consequently, they emphasize the necessity for an intensified investigation into the microenvironmental characteristics of the soil habitat for this species. Field studies have demonstrated that plants thriving in organically rich soil exhibit enhanced vigor and higher population densities (Yang et al., 2017). These findings underscore the imperative for soil microenvironment optimization—particularly organic matter enrichment—combined with scientific irrigation, fertilization, and biotic stress management to reduce cultivation costs and enhance breeding efficiency.

**FIGURE 12:**
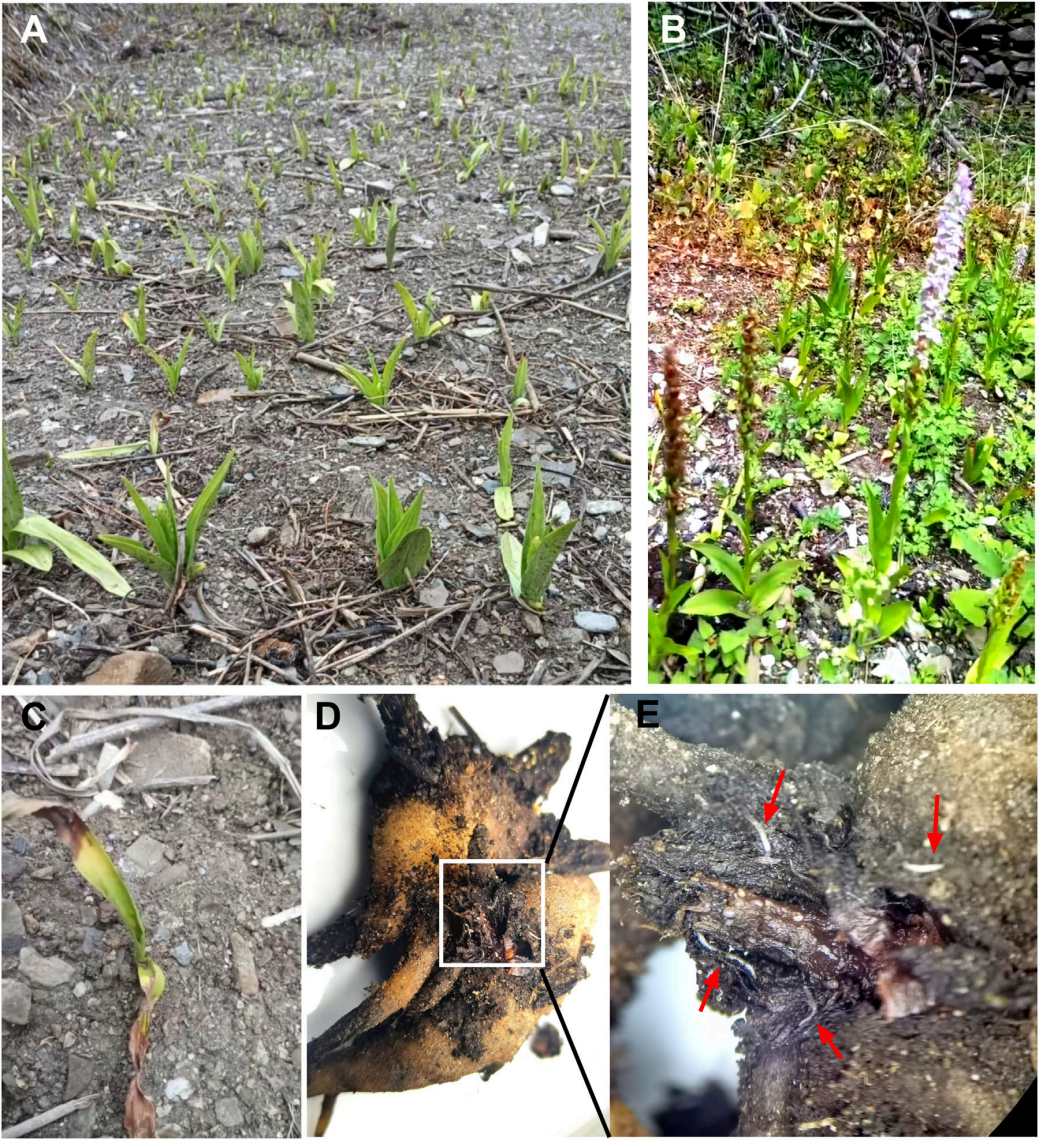
Artificial introduction and cultivation of *G. conopsea*. **(A)** Growth status of plants transplanted in October 2023, documented on 10 May 2024; **(B)** Flowering and fruiting observed on 15 July 2024; **(C)** Plants exhibiting leaf yellowing and desiccation observed on 8 June 2025; **(D)** Decayed tubers from yellowed and desiccated plants; **(E)** Dense colonization of white nematodes in partially decayed tuber tissues (indicated by red arrows). Data sourced from the authors’ original research (unpublished).

In October 2023, we conducted an introduction trial, transplanting wild *G. conopsea* plants from 3,600 m altitude to cultivated fields at 3,200 m. Seedlings emerged in mid-May 2024 but displayed generalized leaf yellowing, potentially linked to transplant injury (Figure 12A). Most plants flowered and fruited normally (Figure 12B). However, by May 2025, many exhibited leaves wilting and mortality (Figure 12C). Excavation revealed root rot and heavy nematode infestation in affected individuals (Figures 12D,E). Field surveys corroborated grower reports of progressive size reduction and population decline in transplanted *G. conopsea*, suggesting nematode-related pathology. Furthermore, most transplants were placed on bare ground with thorough weed removal in soils deficient in organic matter. This likely diminished weed-derived nematode-suppressive exudates and created a suboptimal microenvironment for growth-promoting mycorrhizal fungi (Gai, 2015), leading to malnutrition, weakened resistance, and severe nematode-induced rot. Thus, pre-transplant preparations for *G. conopsea* must include: (1) Soil amendment with organic matter fermented with beneficial mycorrhizal fungi specific to this species. (2) Pre-emptive soil sterilization against pests. (3) Intercropping with nematode-suppressive plants. (4) Installation of shading and humidification systems. These measures will establish a suitable microenvironment for growth. Given current limitations in tissue-culture rapid propagation and seed-based reproduction, strategically integrating vegetative propagation with habitat-mimicking cultivation systems may accelerate breakthroughs in artificial cultivation and ensure sustainable medicinal material supply.

### 9.2 Tissue culture-based rapid propagation

Plant tissue culture technology offers distinct advantages over conventional propagation methods for *G. conopsea*, including accelerated breeding cycles and enhanced multiplication efficiency of elite genotypes (Li et al., 2024). The seeds serve as the quintessential explant materials for tissue culture. While mature seeds from Chinese (Xinglong Mountain, Gansu) and Russian (Novosibirsk) populations exhibit poor germination (Ding et al., 2014; Nabieva et al., 2020), immature seeds demonstrate improved viability (up to 20% germination) (Ding et al., 2014), with embryos at 1–4 months post-anthesis showing optimal germination-seedling transition in PT medium supplemented with 10% potato extract and 1% activated carbon (Gao and Feng, 2012). Notably, Western European ecotypes achieve 40% germination in mature seeds, suggesting geographic genetic divergence in germination physiology (Waes and Debergh, 2010). Seed-based protocols have been refined through optimized media formulations (Table 5): 1/3 MS medium with 0.3 mg/L NAA + 1.0 mg/L 2-iP + 10% coconut water enhances protocorm formation (Nabieva et al., 2020), while 1/2MS medium containing 1.0–2.0 mg/L KT + 0.1 mg/L NAA + 10 mg/L adenine + 20 g/L sucrose + 200 mL/L coconut milk promotes rhizome differentiation via synergistic regulation of cell division (adenine), carbon metabolism (sucrose), and nutrient supply (coconut milk) (Ding et al., 2014). Seed preservation strategies leverage orchidaceous desiccation tolerance, with 4°C storage of dried seeds proving effective for medium-term germplasm conservation, though systematic studies on moisture content and thermal drying impacts remain lacking (Magrini et al., 2019; Jiang, 2022a; Shi, 2023a).

**TABLE 5:** Optimized culture medium formulations and their effects on *G. conopsea* tissue culture.

Basic medium	Organic additives	Hormones	Other additives	Inoculated tissue	Effect/Outcome	Reference
1/2MS	20 g/L Sucrose, 200 m L/L Coconut Milk, 5.0 g/L Agar	1.0∼2.0 mg/L KT, 0.1 mg/L NAA, 10.0 mg/L adenine	1.0 g/L Activated Carbon	Seed	Promote seed germination	Ding et al. (2014)
	20 g/L Sucrose, 200 m L/L Coconut Milk, 5.0 g/L Agar	3.0 mg/L 6-BA, 0.1 mg/L NAA, 10.0 mg/L adenine	1.0 g/L Activated Carbon	Rhizome (callus tissue)	Promote rhizome proliferation and differentiation. High-concentration 6-BA and low-concentration NAA plays a crucial role in rhizome growth and proliferation, but is unfavorable for bud differentiation	Ding et al. (2014)
	20 g/L Sucrose, 200 m L/L Coconut Milk, 5.0 g/L Agar	1.0 mg/L-, KT, 10.0 mg/L adenine	1.0 g/L Activated Carbon	Bud	Promote bud differentiation	Ding et al. (2014)
	20 g/L Sucrose, 6 g/L Agar, 0.5 g/L Casein Hydrolysate	0.5 mg/L NAA	—	Adventitious Bud	Promote root growth with robust root development	Li and Liu (2014)
MS	—	NAA 0.50 mg/L	—	Stem tip	Stem tip with 60% callus induction and minimal browning, while leaves and root tips showed severe browning without callus formation	Li and Liu (2014), Peng et al. (2021b)
	—	0.4 mg/L 6-BA, 0.2 mg/L NAA	—	Shoot tip of *in vitro* plantlet	The induction rate of buds and the subculture proliferation coefficient are both high, which is more conducive to inducing shoot tips to sprout	Gao and Feng (2012)
	—	0.5 mg/L NAA, 0.6 mg/L IBA	1% Activated Carbon	shoot clumps	The effect of root induction is excellent	Gao and Feng (2012)
	20 g/L Sucrose, 6 g/L Agar, 0.5 g/L Casein Hydrolysate	0.1 mg/L TDZ	—	Adventitious Bud	The differentiation rate of callus can be as high as 53.3%	Li and Liu (2014)
	20 g/L Sucrose, 6 g/L Agar, 0.5 g/L Casein Hydrolysate	0.1 mg/L TDZ, 0.1 mg/L NAA	—	Adventitious Bud	It promotes bud formation with robust growth, which is suitable for bud proliferation and growth	Li and Liu (2014)
	7.0 g/L Agar, Sucrose 30 g/L	0.5 mg/L KT, 0.5 mg/L ZT, 1.0 mg/L NAA, 1.0 g/L AC, 2.0 mg/L VC	—	Bud	The effect of inducing buds is relatively good, and the degree of browning is reduced	Yang et al. (2012)
PT	10% *Solanum tuberosu*m Extract	—	—	Embryo	Enhancing germination rate (up to 24%)	Gao and Feng (2012)

Beyond seed explants, Li and Liu (2014) evaluated multiple tissues (young leaves, root meristems, axillary buds, shoot tips, and floral pedicels) for *G. conopsea* micropropagation, identifying shoot tips as optimal with 60% callus induction and minimal browning on MS medium containing 0.5 mg/L NAA (Table 5), while leaves and root tips showed severe browning without callus formation. Yang et al. (2012) developed an integrated anti-browning protocol: sequential sterilization (75% ethanol for 20 s → 0.1% HgCl_2_ for 10 min), 1 g/L activated carbon (higher concentrations suppressed axillary bud growth), and 5–10 days dark pre-culture under 2000 lx light with 3-day subcultures, achieving 60% browning reduction despite 2 mg/L vitamin C’s growth inhibition. Peng K. et al. (2021) confirmed hormonal specificity, showing 0.5 mg/L NAA in MS medium maximized shoot tip callus induction (59.5%), contrasting sharply with 1.0 mg/L 2,4-D’s inefficacy (11.5%), while 6-BA exhibited concentration-dependent effects—0.4 mg/L enhanced clustered bud proliferation (3.8-fold) but >0.4 mg/L reduced subculture capacity by 40% (Table 5; Gao and Feng, 2012). Root induction reached 80% success in 1/2MS + 0.5 mg/L NAA (Li and Liu, 2014). Current limitations necessitate phase-specific media optimization and advanced anti-browning strategies to enable scalable cultivation of this orchid species.

### 9.3 Symbiotic seed germination and seedling cultivation

The dust-like, endosperm-deficient seeds of *G. conopsea* exhibit obligate mycoheterotrophic germination, relying exclusively on symbiotic fungal colonization (typically *Ceratobasidiaceae* or *Tulasnellaceae*) for nutrient acquisition in natural ecosystems (Shi L. et al., 2022; Yao et al., 2024). These mycorrhizal partners supply critical resources—water, micronutrients, phytohormones, and antimicrobial compounds—that simultaneously suppress competing microbes and activate embryonic development (Stark et al., 2009; Steinfort et al., 2010). However, the stochastic distribution of compatible fungi in soil matrices, combined with limited seed dispersal efficiency, creates ecological bottlenecks, resulting in <1% natural germination success and severely constraining population recruitment (Mccormick et al., 2018; Li T. et al., 2021). To address this, targeted isolation of germination-promoting fungi has emerged as a key strategy for enhancing propagation efficiency. Xing et al. (Gao et al., 2019) pioneered this approach by identifying *Ceratobasidium* sp. GS2 from root endophytes, demonstrating its capacity to drive protocorm formation and seedling development via *in situ* seed-fungus co-cultivation (Table 6; Yue, 2020). Fungal specificity studies reveal narrow symbiotic compatibility: among 102 isolates from protocorms and seedlings, only *Ceratobasidiaceae* strains supported full germination-to-seedling transitions, while *Schizophyllaceae*, *Irpicaceae*, and *Polyporaceae* showed negligible efficacy, suggesting ecological niche partitioning (Jiang et al., 2022b). Table 6 summarizes the main endomycorrhizal fungal strains implicated in studies on the symbiotic germination of *G. conopsea* seeds and their effects in promoting seed germination and growth.

**TABLE 6:** Fungal strains associated with *Gymnadenia conopsea* and their effects on seed germination.

Strain	Genus	Family	Germination-promoting effect	Reference
GS2	*Ceratobasidium* sp.	*Ceratobasidiaceae*	Supports protocorm formation and seedling development via symbiotic co-cultivation	Gao et al., 2019; Shi (2023a)
—	*Tulasnellaceae* spp.	*Tulasnellaceae*	Dominant symbionts in European populations; essential for nutrient acquisition during germination	Gao et al. (2020), Xing et al. (2020)
GB32	*Tulasnellaceae* sp.	*Tulasnellaceae*	The development halts at the protocorm stage, and cannot support further progression to the seedling stage	Gao et al. (2020)
GB1	*Tulasnellaceae* sp.	*Tulasnellaceae*	Cannot promote seed germination through to the seedling stage	Gao et al. (2020)
—	*Irpicaceae* spp.	*Irpicaceae*	No observed germination support	Jiang (2022a)
	*Polyporaceae* spp.	*Polyporaceae*	Ineffective in promoting germination	Jiang (2022a)

Geographic divergence in mycorrhizal partnerships further complicates propagation strategies. European *G. conopsea* populations predominantly associate with *Tulasnellaceae* fungi, whereas Asian ecotypes rely on *Ceratobasidiaceae* symbionts, reflecting potential co-evolutionary adaptations to regional soil microbiomes (Xing et al., 2020). This biogeographic specificity underscores the necessity for location-tailored fungal isolation protocols. For instance, Xing’s strain-mixing technique achieved preliminary success in naturalized germination but requires refinement for cross-regional applicability (Yue, 2020). Current limitations in scalable symbiotic systems highlight unmet needs: 1) systematic screening of germination-active fungi across diverse habitats, 2) optimization of the symbiotic germination system between fungi and seeds, and 3) elucidation of molecular mechanisms governing fungal recognition and nutrient exchange. Addressing these gaps will enable engineered symbiotic germination platforms to bypass natural recruitment bottlenecks, facilitating large-scale conservation and cultivation of this ecologically vulnerable orchid.

## 10 Conclusion and future prospects

*G. conopsea* has emerged as a critically important medicinal resource, validated by its ethnopharmacological legacy, phytochemical richness, and diverse pharmacological activities. Phytochemical studies have identified 203 bioactive compounds, including benzyl ester glucosides, stilbenoids, and polysaccharides, which collectively underpin its anti-oxidant, immunomodulatory, neuroprotective, and anti-fatigue properties. Despite these advancements, pharmacological researches remain largely limited to crude aqueous/alcoholic extracts (Table 3). Few isolated compounds (Tables 1, 4) have subjected to systematic *in vitro* bioactivity validation, and *in vivo* pharmacological testing is notably scarce. Consequently, the mechanistic links between specific compounds and their therapeutic effects remain inadequately derexplored, impeding the development of standardized preparations and quality control protocols.

Current literature indicates that *G. conopsea* polysaccharides are abundant and exhibit immunomodulatory, tonic, and anti-fatigue activities. However, critical knowledge gaps persist regarding their branching structures, *in vivo* absorption/distribution, and molecular targets. Benzylester glucosides—present at high levels in alcoholic extracts—demonstrate neuroprotective effects consistent with the *G. conopsea*’s documented enhancement of memory and cognitive function. Nevertheless, these compounds lack validation in animal models and identification of specific molecular targets. Despite being the most abundant bioactive constituents, standardized studies investigating potential synergistic interactions between polysaccharides and benzylester glucosides are exceptionally limited. In antioxidant research, *in vitro* evaluations have primarily focused on free radical scavenging capacity, which shows minimal correlation with *in vivo* animal studies demonstrating modulation of endogenous antioxidant enzyme systems. Future work should employ validated cellular models to identify active metabolites responsible for upregulating antioxidant enzymes. For antiviral screening, expanded use of viral molecular target models and advanced techniques—such as reporter gene systems, surface plasmon resonance (SPR), protein microarrays, and molecular docking—is essential. Given that viral infections trigger excessive immune responses (e.g., cytokine storms), immune dysfunction, and multi-organ failure, botanical medicines offer advantages through their multi-component/multi-target nature and synergistic interactions. These properties support efficacy in modulating inflammatory responses, mitigating tissue damage, and alleviating symptoms (e.g., cough, fatigue) (Bai et al., 2025), providing strategic directions for evaluating *G. conopsea*’s antiviral potential and elucidating its active metabolites and mechanisms.

Progress in pharmacological research and clinical applications of *G. conopsea* is contingent upon sustainable raw material supply. However, wild resources are nearly depleted, and artificial cultivation remains unrealized—a challenge demanding urgent technological innovation. Current cultivation systems are immature, with domestication efforts hampered by unresolved bottlenecks such as progressive dwarfism and yield reduction in successive plantings. Key priorities include addressing nematode infestations and establishing a conducive microenvironment for mutualistic growth between *G. conopsea* and its symbiotic mycorrhizal fungi. Tissue culture faces challenges including tissue browning, low multiplication rates, protracted culture cycles, and high costs. Optimization requires refined culture media formulations to improve proliferation efficiency and reduce browning incidence, alongside automation to lower production costs. Symbiotic germination represents a promising solution for resource scarcity but necessitates further screening for high-efficiency endophytic fungal strains and optimization of symbiotic seedling systems (e.g., substrate composition, environmental controls) to enable scalable seedling production and ultimately resolve critical supply limitations.
